# Temperature-Dependent Rotation of Protonated Methyl Groups in Otherwise Deuterated Proteins Modulates DEER Distance Distributions

**DOI:** 10.1007/s00723-024-01720-5

**Published:** 2024-10-03

**Authors:** Thomas Schmidt, Valentyn Stadnytskyi

**Affiliations:** Laboratory of Chemical Physics, National Institute of Diabetes and Digestive and Kidney Diseases, National Institutes of Health, Bethesda, MD 20892-0520 USA

## Abstract

Temperature-dependent DEER effects are observed as a function of methyl rotation by either leucine- or nitroxide-specific protonated methyl groups in an otherwise deuterated background. Both species induce a site-specific enhancement in the apparent *T*_m_ relaxation of the paramagnetic nitroxide label. The presence of a single protonated methyl group in close proximity (4–10 Å) to only one of the two nitroxide rotamer ensembles in AviTagged immunoglobulin-binding B domain of protein A results in a selective and substantial decrease in *T*_m_, manifested by differential decay of the peak intensities in the bimodal *P(r)* distance distribution as a function of the total dipolar evolution time, temperature, or both. The temperature-dependent differential decay of the individual distance components was globally analyzed by fitting the DEER dipolar time traces to a three-site jump model that is defined by the activation energy of leucine- or nitroxide-specific methyl rotation. Temperature-assisted T_m_ filtering will capture the DEER structural analysis of biomolecular systems heterogenic conformations, including complexes involving multimeric proteins.

**Supplementary Information:**

The online version contains supplementary material available at 10.1007/s00723-024-01720-5.

## Introduction

**Fig. 1:**
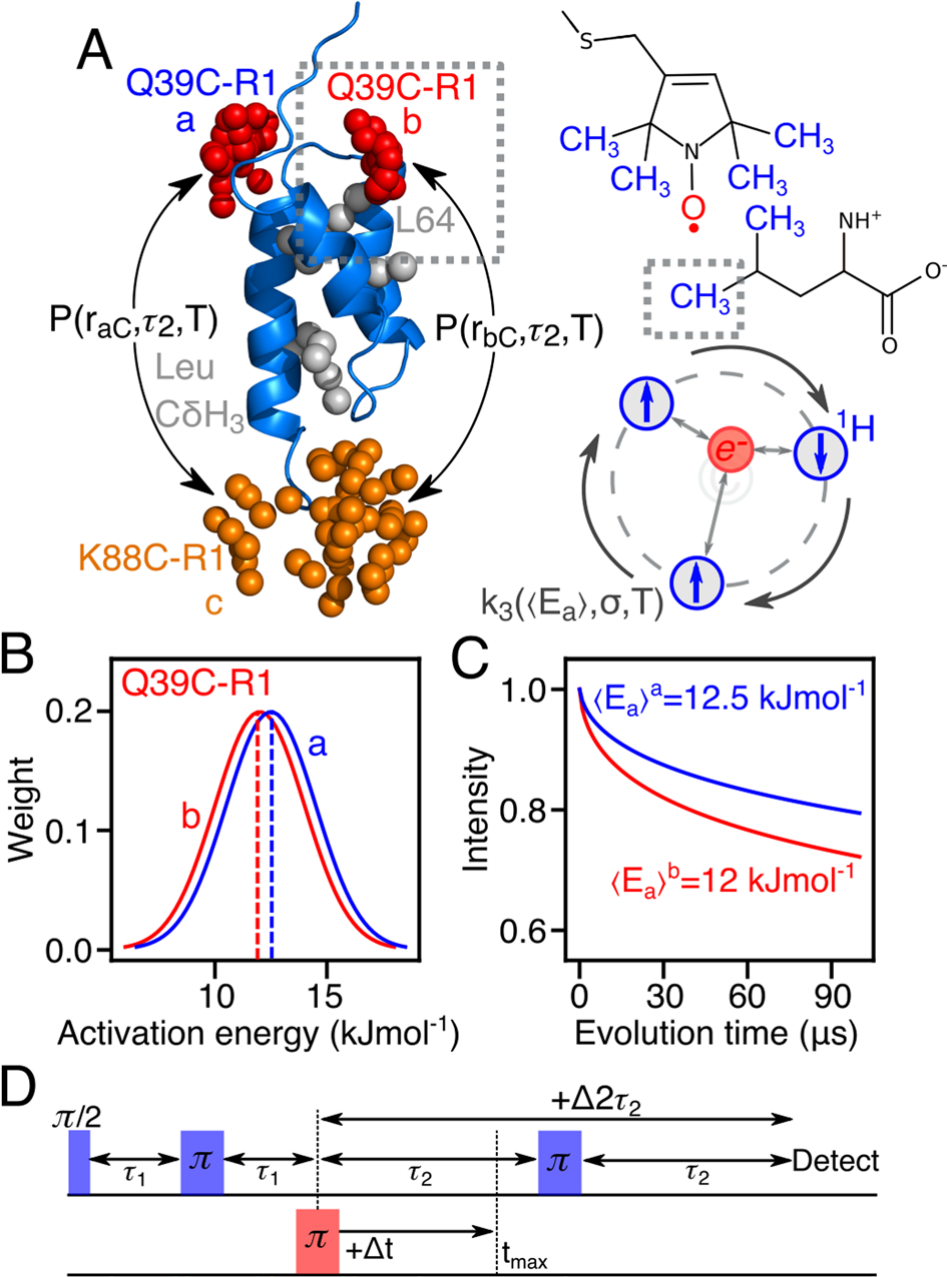
Temperature-dependent *P(r)* modulation derived from rotation of protonated methyl groups of the leucine or nitroxide moieties. **A** Ribbon diagram of the protein A core domain (*blue*) with the oxygen atoms of the nitroxide spin label ensembles (generated using the rotamer library program MMM) colored in red (Q39C-R1) and orange (K88C-R1), and the leucine methyl groups in grey. The two distances distribution (P) between K88C-(R1) and the two conformer populations (*a* and *b*) of Q39C-R1 are indicated as a function of distance (*r*), evolution time (*t*_max_), and temperature (T). The grey box indicates the rotamer b electron-Leu64 proton hyperfine coupling of interest and is presented. MTSL rotamer b of Q39C-R1 with hyperfine coupled Leu64; the protonated methyl groups in close proximity to the electron bearing oxygen (*red*) is presented in blue, which is also shared with the bonded nitrogen. The methyl three-site jump for a protonated methyl group coupled to an electron (*red*) as a function of mean activation energy, 〈*E*_a_〉, and its distribution (σ); the proton spin states are indicated by blue arrows without the influence of spin diffusion. **B** Simulated activation energy (*E*_a_) and its distribution (1.5 kJmol^−1^) was generated by Eq. 1 set to 12 and 12.5 kJmol^−1^. **C** Shows the weighted three-site jump rate using Eq. S2 that ultimately gives rise to the modulated transverse relaxation factors. **D** The *T*_m_-edited DEER experiment records the dipolar time trace as function of both evolution time (Δ*T*) and dipolar evolution time increments (Δ*t*)

Pulsed double electron–electron resonance (DEER) EPR spectroscopy provides means to accurately measure long-range distances between pairs of paramagnetic labels up to 170 Å under deuterated conditions, and therefore is an invaluable tool for conformational analysis of proteins [1–3]. The 4-pulse DEER experiment (Fig. S1) produces a Hahn-echo at the observer frequency with an electron–electron distance modulation introduced by an ELDOR pulse at the pump frequency; however, the overall echo intensity largely depends on the electron phase-memory relaxation time, *T*_m_ [4, 5]. The *T*_m_ is anticorrelated to the number of near protonation sites; thus, protonated moieties (e.g., methyl groups) in close vicinity to the spin label decrease the DEER echo [6, 7]. The model protein, AviTag-Protein A (Fig. 1A), has the MTSL spin label (R1) attached to engineered cysteine residues (Q39C/K88C) within the ordered Protein A domain [18]. Previous measurements showed two resolved distances in the P(r) distribution at 33 and 38 Å, arising from the Q39C-R1 label occupying two distinct regions of the conformational space (respectively labeled a and b in Fig. 1A), as judged by the predicted P(r) distribution generated from the atomic coordinates (PDB: 1bbd [18]) using the spin-label rotamer program MMM, ChiLife or Xplor-NIH (Fig. S2) [19–21]. The “true” bimodal distribution can be isolated in a deuterated system presenting the cumulative distances between individual MTSL rotamers; however, protonation of Leu64 or MTSL resulted in dramatic changes in the apparent distance population as observed by *T*_m_-edited DEER. Here, protonated methyl group(s) attached to either the leucine sidechain (less than 10 Å from the electron) or the paramagnetic label itself in a deuterated background permits the acquisition of a pseudo-3D DEER spectrum via the *T*_m_-edited DEER experiment [8]. *T*_m_-edited DEER is an implementation of the 4-pulse DEER experiment in which both the dipolar evolution time (*t*_max_) and the evolution time (*τ*_2_) are incremented (Fig. 1D), therefore modulating the DEER distance populations as a function of evolution time. The increased dimensionality, comprising temperature (T), *T*_m_ relaxation (*τ*_2_ period), and dipolar evolution time (*t*_max_), permits resolving multimodal distance distributions as in proteins displaying several labeling sites, oligomeric complexes or conformational heterogeneity [9, 10]. This approach relies on the chemical environment-dependent methyl rotation and their unique activation energy barriers (*E*_a_) that subsequently modulates the *T*_m_ of paramagnetic spins specific to their molecular origin (Fig. 1A) [11–17].

The rationale for the *T*_m_-edited DEER-derived methyl rotation activation energy barriers (*E*_a_) is shown in Fig. 1 based on previous work established by Vugmeyster et al. [12]. Here, two individual paramagnetic rotamers where one of which is in proximity to protonated methyl moieties (leucine or R1) in an otherwise deuterated environment experience activation energy barriers (*E*_a_), in Fig. 1B this was simulated using an estimated value of 12 and 12.5 kJmol^−1^ with a distribution of 1.5 kJmol^−1^ [12, 13]. This *E*_a_ distribution will give rise to a weighted three-site jump rate (*k*_3_) using Eq. S3 as a function of temperature, which ultimately modulates the apparent *T*_m_. While the individual paramagnetic rotamers experience a difference in their *T*_m_ decay rate, that translates to a differential decay of their weighted dipolar couplings, or the population of their individual distance peaks at 32 and 38 Å. Therefore by taking the population ratio (*P*_bc_/*P*_ac_) of peaks at 32 and 38 Å at each temperature the DEER experiment was recorded, one can extract the difference between the individual activation energies in relation to the applied temperature.

## Results and Discussion

Previous measurements on protein A have shown that upon protonation of Leu64 or MTSL resulted in dramatic changes in the apparent distance population as observed by *T*_m_-edited DEER. Here we extended the expression to include the modulation factor *k*_app_ capturing methyl group rotation as a function of temperature [4]. The chemical origin of the relaxation components was disentangled through site-specific protonation of either MTSL and Leu64 methyl groups (Fig. 1B). The three-site jump model predicts the longitudinal relaxation rate for methyl groups based on the jump constant *k*_3_ that distinguishes the methyl conformers by their unique values of activation energy barrier (Fig. 1C).

**Fig. 2:**
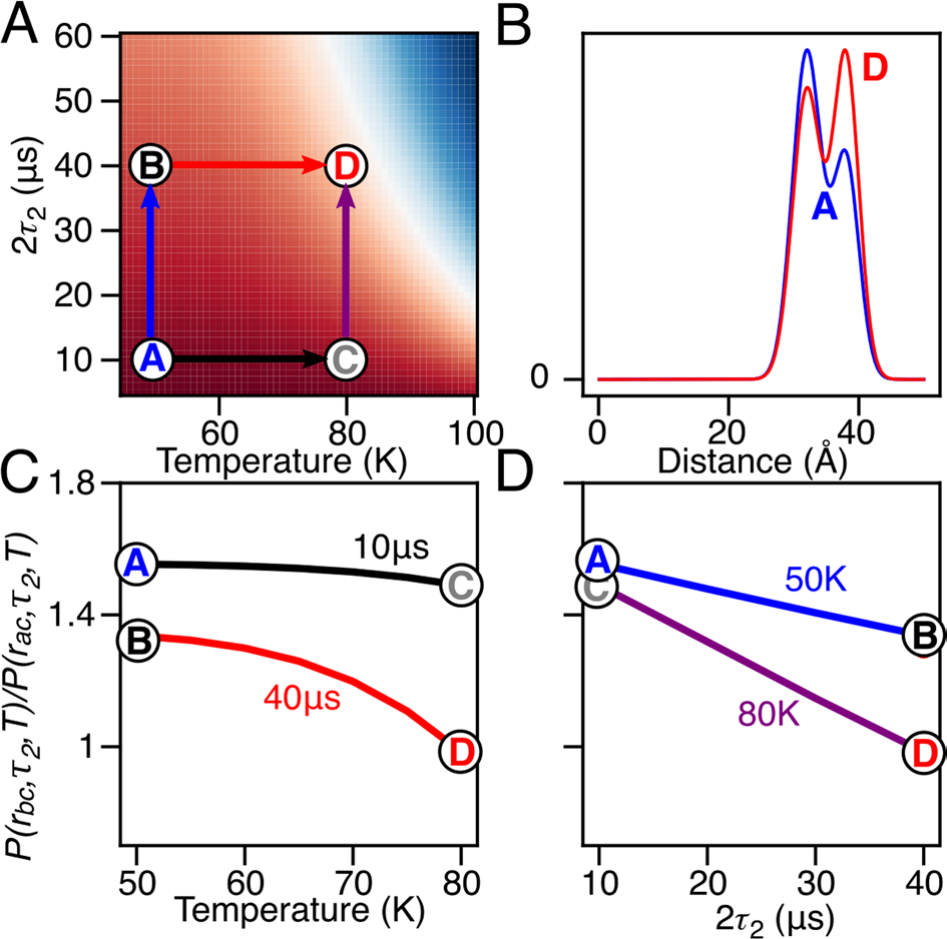
Simulated distance distribution ratio. **A** Heatmap shows the distance population ratio as function of relaxation time (2*τ*2) and temperature (T) ranging between 1.6 (*red*) to 0 (*blue*). The heatmap was simulated with *k*_0_ = 7 × 10^11^ s^−1^, *E*^*a*^_*a*_ = 12 kJmol^−1^, *E*^*b*^_*a*_ = 12.5 kJmol^−1^, *σ*^a^ = 2.5 kJmol^−1^, *σ*^b^ = 2.5 kJmol^−1^, *T*^*a*^_*m*_ = 42 μs, *T*^*b*^_*m*_ = 28 μs and *P*_bC_/*P*_aC_ = 0.62. Letters designate individual DEER distance distribution for *A*(10 μs, 50 K, *blue*), *B*(40 μs, 50 K, *black*), *C*(10 μs, 80 K, *lilac*) and *D*(40 μs, 80 K, *red*). **B** Comparison between DEER distance distribution observed for state *A* to state *D* shows population decrease at 32 Å compared to 38 Å. **C** presents population ratio changes as a function of temperature along the transition path of the temperature dimension for states *A*-to-*C* (black) and *B*-to-*D* (red). **D** The transition path along the 2*τ*_2_ time dimension for states *A*-to-*B* (blue) and *C*-to-*D* (lilac)

The ratio of the *b* to *a* components in the *P(r)* distributions, P(r_bC_,τ_2_,T)/P(r_aC_,τ_2_,T), for [Leu-CH_3_/^2^H]-AviTag-Protein A (Q39C-R1/K88C-R1) shown in Fig. 1, is expected to decay as:

1 $$\frac{{P(r_{bc} ,\tau_{2} ,T)}}{{P(r_{ac} ,\tau_{2} ,T)}} = \frac{{p_{b} }}{{1 - p_{b} }}\exp \left( { - 2\tau_{2} \frac{{R_{m}^{b} /k_{\text{app}}^{b} - R_{m}^{a} /k_{\text{app}}^{a} }}{2}} \right),$$

where *p*_*b*_ is the Q39C-R1 nitroxide population in the *b* state, and *R*^*b*^_*m*_ (= 1/*T*^*b*^_*m*_) and *R*^*a*^_*m*_ (= 1/*T*^*a*^_*m*_) are the phase-memory relaxation rates for Q39C-R1 in the *b* and *a* states (Fig. 2) [4]. While echo decays of nitroxides are rarely monoexponential, here we apply this simplification to isolate differences between the individual relaxation rates which are introduced with increased temperature. Interestingly, the rotamer specific phase-memory relaxation rates are modulated by *k*^a^_app_ and *k*^b^_app_, respectively, where *k*_app_ is a rotamer specific modulation of *T*_*m*_ that depends on the rotamer specific activation energy (*E*_*a*_) described in Eq. S4 that depends on localized chemical environment (e.,g. charge, hydrophobicity and hydrogen bonding) [12, 13]. Further, the temperature insensitive *T*_*m*_ (at the regime between 20 and 85 K) is potentially established through means of spin flip-flopping. While methyl group tunneling is known to induce electron echo decay, here we harness the difference to differentiate distance distributions. While both labeling sites are influenced by methyl rotation, the DEER data only present the difference thereof, hence it is practical to present the relaxation difference from the frame of the slowest relaxing electron by setting *k*^*a*^_app_ to 1 and k_0_ to the same for all conformers. Further, the electron *T*_*m*_ decreases with increasing temperature, the presented analysis permits to maintain the same *T*_*m*_ for all temperatures (42 μs [4]) based on a fully deuterated sample.

Figure 2 highlights the *P(r)* relationship between the 2τ_2_ time interval and the temperature induced methyl rotation as outlined by Eq. 1; the induced relaxation pathway shifts the apparent *P(r)* conformation from state *A* (blue) to *D* (red) by *T*_*m*_ modulations at 80 K with a 2*τ*_2_ of 40 μs (Fig. 2B). The individual changes in *P(r)* ratio along the *T*_*m*_ relaxation pathway due to either temperature or 2*τ*_2_ are outlined in Figs. 2C and S3D, respectively. Overall, Fig. 2 highlights the selection of values of temperature and 2*τ*_2_ to achieve the desired *P(r)* ratio based on localized protonated methyl groups and their rotation.

**Fig. 3:**
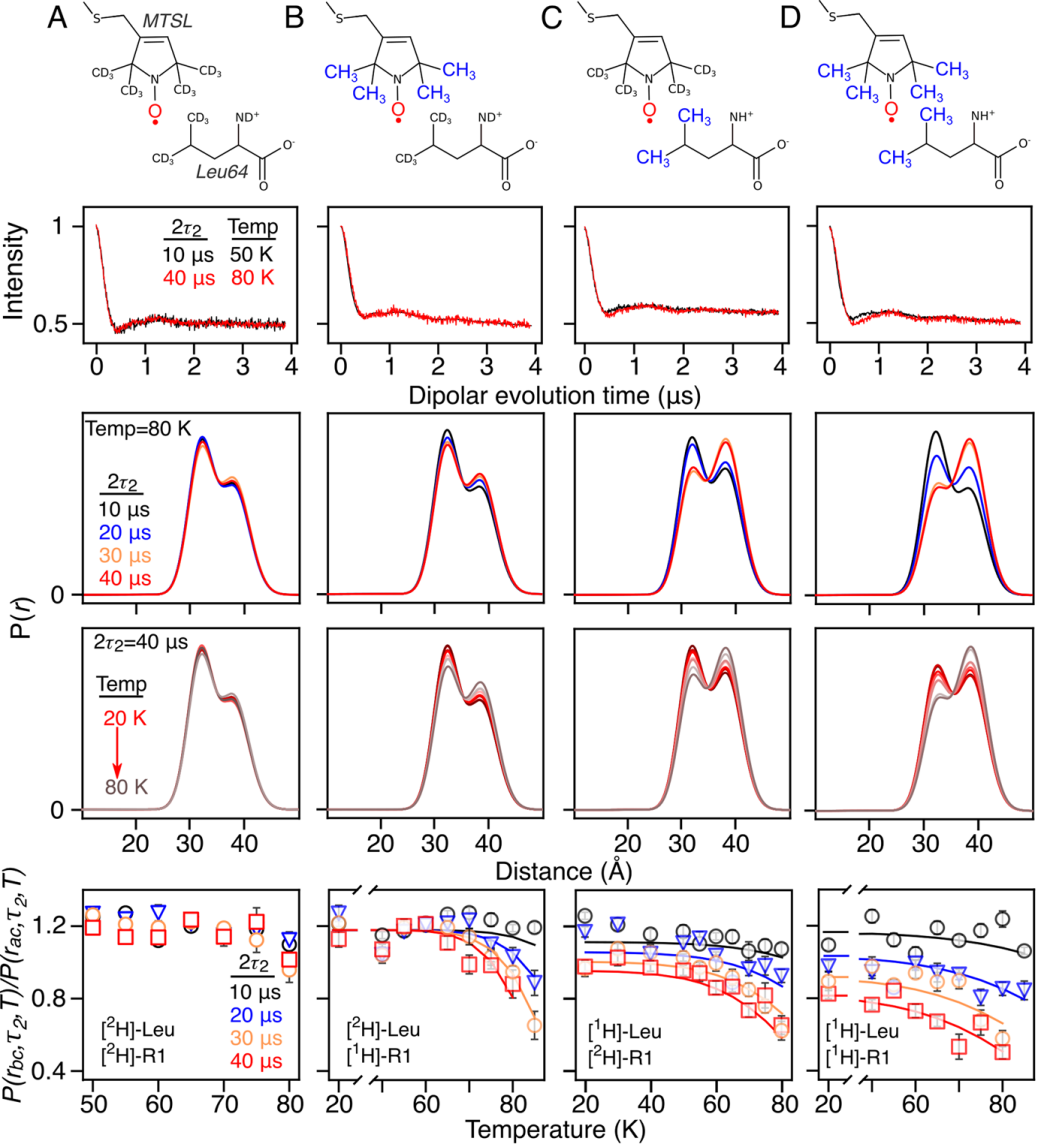
Rotation of protonated leucine methyl groups in conjunction with spin diffusion promote transverse relaxation as probed by selective protonation of MTSL and leucine moieties. Fully deuterated protein A (Q39C/K88C) was covalently linked to either **A** deuterated or **B** protonated R1 label while Leu64 remained deuterated, subsequently protonated Leu63 was conjoined with **C** deuterated or **D** protonated MTSL to detangle the chemical origin of *Tm* relaxation. The electron bearing oxygen is shown in red while protonated methyl groups are shown in blue, the leucine 64 remained deuterated in cases A and B but was protonated for cases **C** and **D**. The middle rows depict the bimodal distribution obtained from Gaussian fitting using the mean distribution and width as global parameters. The peak intensity at ~ 32 Å, corresponding to the *P(r*_*bC*_*)* in Fig. 1, decreases because the increased methyl rotation occurs concomitantly with increasing temperature. The coloring scheme refers to the 2τ2 set to either 10 (*black*), 20 (*blue*), 30 (*orange*), or 40 μs (*red*) in the T_m_-edited DEER experiment. The distance distribution obtained by validated Tikhonov regularization are shown in Figs. S3–S4, S7–S8, S13–S14, and S19–S20 for columns (A), (B), (C) and (D), respectively. In the lower row, changes in distance population ratios, *P*(*r*_bc_,*τ*2,*T*)/*P*(*r*_ac_,*τ*2,*T*), are distinguished by *T*_*m*_-edited DEER with 2*τ*2 set to either 10 (*black*), 20 (*blue*), 30 (*orange*), or 40 μs (*red*) at temperatures ranging between 20 and 80 K. Hollow points present population ratios obtained by global Gaussian modeling with two Gaussians in which peak positions and widths are treated as global parameters without restraints in amplitude to the dipolar evolution time domain; the individual fits are shown in Figs. S6–S7, S10–S11, S16–S17, and S22–S23 for columns (A), (B), (C), and (D), respectively. In contrast, the curves were fit to the dipolar evolution time domain with additionally amplitudes restrained to Eq. S2 and hence dependent on the evolution time (2*τ*2) and rotation (*k*_3_) of the leucine and MTSL protonated methyl groups; the individual fits are shown in Figs. S12–S13, S18–S19, and S24–S25 for columns (B), (C), and (D), respectively. Note that the total area under the *P*(*r*) distribution is always normalized to 1 at every point in the titration. The reduced *χ*^2^ and global parameters of the fits are provided in Tables 1 and S1. The complete temperature/*τ*_2_ titration dataset and analysis is provided in SI Appendix

Data collection was performed in a pseudo three-dimensional fashion whereby temperature, 2*τ*_2_ interval, and dipolar evolution time serve as independent parameters. The conjoint fitting of those variables describes the relaxation contributions by protonated methyl groups attached to either label, leucine, or the combination thereof. Here, the following isotope labeling schemes were tested: ^2^H-MTSL/^2^H-Leu64 (Fig. 3A), ^1^H-MTSL/^2^H-Leu64 (Fig. 3B), ^2^H-MTSL/^1^H-Leu64 (Fig. 3C), and ^1^H-MTSL/^1^H-Leu64 (Fig. 3D). In Fig. 2A, the *T*_m_-edited DEER experiment showed only minor changes in the distance population ratios for all set values of evolution time and temperature for fully deuterated protein A covalently linked to deuterated MTSL [7, 22]. This is not surprising as differences in the Tm due to spin diffusion and methyl rotation are expected to be negligible upon substitution of protons with deuteriums. In comparison, the addition of protons to the MTSL label affected the distance distribution at temperatures above 60 K (Fig. 2B); the population ratios decreased with increasing temperature and evolution time originating from induced methyl rotation. DEER population ratios at 2*τ*_2_ set to 10 μs did not present significant variations for all temperatures; however, upon increasing the 2*τ*_2_ to longer evolution times, the ratio decreased with increasing temperature to a minimum ratio of 0.6 at 85 K and a 2*τ*_2_ of 40 μs. Methyl rotation, experienced by Q39C-R1 nitroxide rotamer *a* and *b,* was fit to the dipolar time traces by Eq. 1, while varying the activation energy, < *E*_*a*_ >, and its distribution, σ. The apparent < *E*_*a*_ > of 11.9 kJmol^−1^ for the *b*-rotamer is induced by chemical environment-dependent effects on methyl rotation of the MTSL rotamer population, such as steric hindrance and hydrophobicity at various labeling sites.

**Table 1:** Isotope labeling schemes and their fitted values including *T*_*m*_ values obtained by measuring for the *b*-rotamer population

protein A	MTSL	< *E*_*a*_ > (kJmol^−1^)	*σ*_<Ea>_ (kJmol^−1^)	*T*^*b*^_*m*_ (μs)	*P*_*bc*_/*P*_*a*c_	*χ*^2^
^2^H	^2^H	–	–	–		1.1^#^/1.2^*^
^2^H	^1^H	11.9 ± 0.1	1.1 ± 0.2	–	0.54 ± 1	1.0^#^/1.3^*^/1.3^✝^
^1^H-Leu-CH_3_/^2^H	^2^H	12.5 ± 0.5	3.5 ± 0.5	29.3 ± 0.5	0.54 ± 1	1.4^#^/1.4^*^/1.4^✝^
^1^H-Leu-CH_3_/^2^H	^1^H	13.4 ± 0.2	3.6 ± 0.4	20.9 ± 0.4	0.55 ± 1	1.1^#^/1.1^*^/1.2^✝^

^#^Averages for model-free fits to the individual DEER echo curves using DeerLab with validated Tikhonov regularization (*n* = 1000)

*Gaussian global fits based on the program DD/GLADDvu, incorporated into an in-house Python program to the complete set of Q-band DEER echo curves recorded over a series of temperate and *τ*_2_ values. For the presented two-Gaussian global fit, only the mean distances and corresponding widths are constrained to be invariant

^✝^In the two-Gaussian global fit, the dependence of the fractional populations of the distance peaks on temperature and τ_2_, as specified by the three-site jump rate and the resulting weighted exponentials as in Eq. S4 (Fig S2), was included as an optimized parameter

Localized *T*_*m*_ relaxation can be modulated by introducing amino-acid-specific protonation, subsequently adjusting it via spin diffusion and methyl rotation of the proton-bearing amino acid [4]. Here, protonated leucine methyl groups reduced the relative DEER distance population inversely related to 2*τ*_2_ and temperature in the *T*_*m*_-edited DEER time traces. Figure 2C depicts deuterated MTSL in the vicinity of protonated leucine methyls; the temperature-driven rotation of the methyl groups decreased the distance ratios with increasing evolution time. In contrast to the previous case (Fig. 3A, B), spin diffusion presents a temperature-independent relaxation component of the b-rotamers (*T*^*b*^_*m*_ = 29 μs); hence, 2*τ*_2_-associated population ratios will not converge at low temperatures (40 K). In the presented fits, activation energy for the leucine methyl groups is 12.5 kJ mol^−1^ with a distribution of 3.5 kJmol^−1^, the increase in *σ*_<Ea>_ compared to previous values originates from the Leu64 methyl groups in vicinity to the MTSL. Protonation of both MTSL and leucine is conjoined in Fig. 2D; the spin diffusion rate is enhanced due to the increase in coupled protons in close vicinity (< *E*_*a*_ >  = 13.4 kJmole^−1^) with no change in *σ*_<Ea>_. The similarities to ^2^H-MTSL/^1^H-Leu64 present the dominance the leucine methyl groups exhibit over the nitroxide relaxation behavior. Overall, we present evidence that amino acid methyl causes both spin diffusion and methyl rotation.

**Fig. 4:**
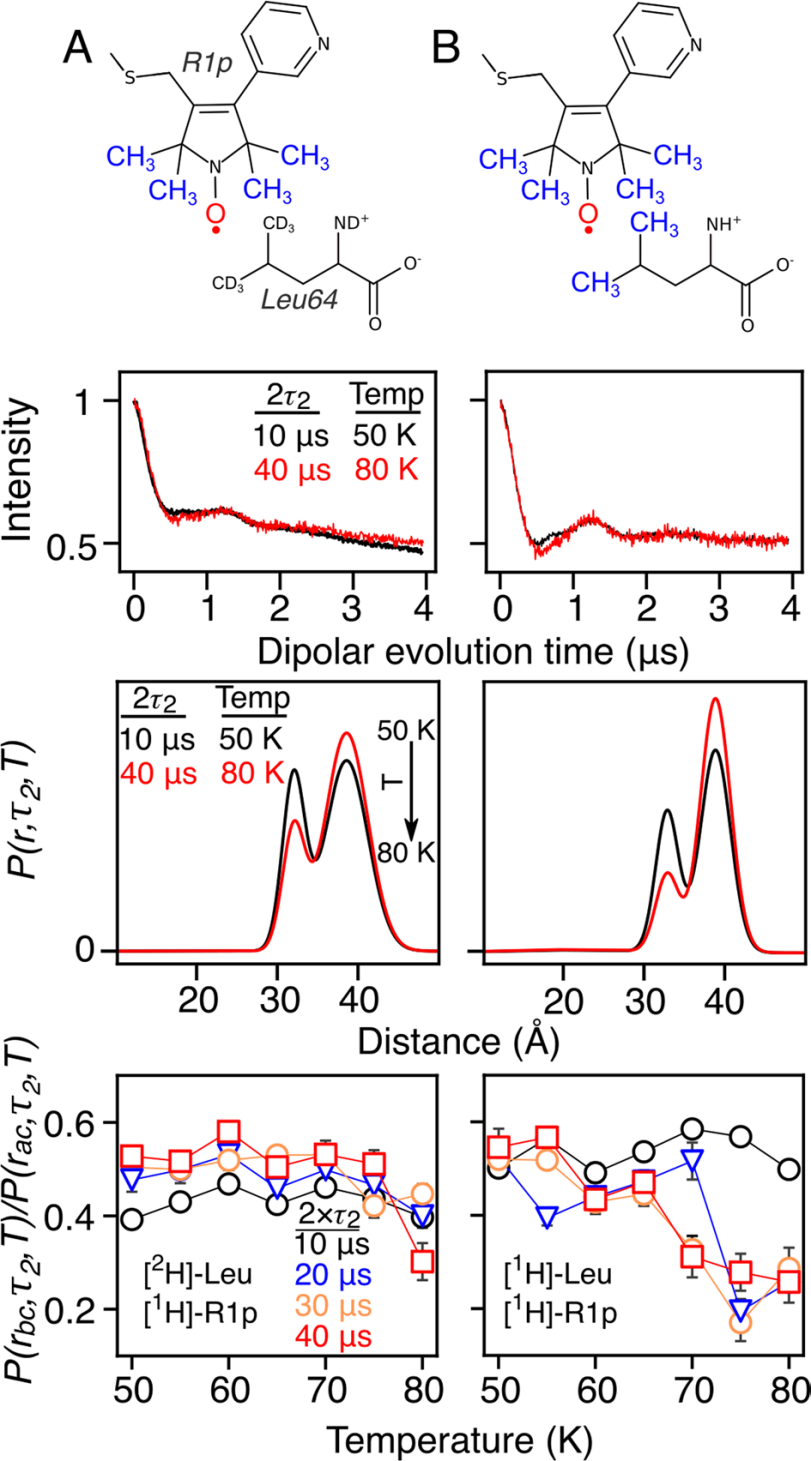
The protein A (Q39C-R1p/K88C-R1p)-related distance population presents a dependence on 2τ_2_ and temperature due to spin diffusion and methyl rotation, respectively. Fully deuterated protein A (Q39C/K88C) was covalently linked to protonated R1p label, while Leu64 was either **A** deuterated or **B** protonated. The electron-bearing oxygen is shown in red, while protonated methyl groups are shown in blue. Changes in distance population ratios, *P*(*r*_bc_,*τ*_2_,*T*)/*P*(*r*_ac_,*τ*_2_,*T*), are distinguished by *T*_*m*_-edited DEER with 2*τ*_2_ set to either 10 (*black*), 20 (*blue*), 30 (*orange*), or 40 μs (*red*) at temperatures ranging between 50 and 80 K. The distance distribution obtained by validated Tikhonov regularization are shown in Figs. S25–S26 and S29–S30 for columns (A) and (B), respectively. Hollow points present population ratios obtained by a global fit to the dipolar evolution time domain that maintained mean distance and width of distribution without restraints in the amplitude; The distance distribution obtained by two-Gaussian fits are shown in Figs. S27–S28 and S31–S32 for columns (A) and (B), respectively. The reduced *χ*^2^ and global parameters of the fits are provided in Table S2

R1p is similar to MTSL with the addition of the 4-pyridyl which increases its size; hence, presents a narrower rotamer distribution [24]. Here, the R1p nitroxide label presented a similar effect on *T*_*m*_ relaxation as a function of temperature and 2*τ*_2_ on the DEER distance distribution. In Fig. 4A, protonated R1p label is in a fully perdeuterated environment, i.e., upon increasing the temperature, a dispersion in population height marks a temperature effect; however, due to the large population difference, the ratio change is rather small. Leu64 was protonated in addition to the R1p label in Fig. 4B; the protonated methyl groups increased susceptibility to spin diffusion and temperature which was similar to the difference between 10 and 40 μs DEER traces with increasing temperature. We did not attempt to fit the resulting DEER traces, as it is unknown how the pyridyl group contributes to observed spin diffusion and temperature effects. Interestingly, previously Bahrenberg et al. [23] reported that under certain conditions, the signal can increase with increasing τ_2_, which is similar to what is seen for R1p (Fig. 4A). While the leucine methyl group exhibited similar temperature/2*τ*_2_ dependencies as observed for the MTSL samples, the extent of the modulation converges between 70 and 80 K that differs from MTSL. Therefore *T*_*m*_/temperature-modulated DEER provides means differentiate alternative paramagnetic labels (e.g., R1p).

## Conclusion

In summary, temperature titration of nitroxide-labeled proteins distinguishes multimodal distance distributions by DEER spectroscopy. We characterized the relationship between temperature-induced methyl rotation and differential relaxation of the paramagnetic labels that ultimately modulate a bimodal *P(r)* distribution in AviTag-Protein A as proof of principle. Conjoined titration of temperature and evolution time resulted in apparent populations of two DEER distance distributions modulated by rotational diffusion of protonated methyl group(s) to either protein sidechain (less than 10 Å from electron) or even the paramagnetic label itself in an otherwise fully deuterated background. Here, the localized chemical environment of individual protonated nitroxide labels allows temperature-driven spin differentiation without sidechain protonation which is enhanced upon the introduction of protonated leucine moieties (L64). Vast applications toward protein complexes and their equilibrium kinetics are foreseen; for example, structural information on an equilibrium of homodimeric proteins will present multiple spin labels; therefore, the assignment of DEER distance distribution to their paramagnetic centers is essential. While *T*_*m*_-edited DEER in conjunction with selective protonation is used to separate individual DEER distances, the introduction of localized methyl labels in a deuterated background is cumbersome; therefore, the use of protonated nitroxide labels in conjunction with temperature titration forms an alternative to assign distance distribution in oligomeric systems. Lastly, DEER distance distribution produced by orthogonal labeling with different nitroxide labels can be distinguished based on temperature titrated *T*_*m*_-edited DEER data due to their chemical specific methyl rotation. Overall, temperature titration and *T*_*m*_-edited DEER in conjunction with site-specific protonation expands the repertoire to assign DEER distances in oligomeric biomolecular systems.

## Methods

### Expression, Purification, and Labeling of Protein A

Fully deuterated AviTag-protein A, with two surface exposed, engineered cysteine residues (Q39C and K88C) was expressed in *E. coli* and purified as described previously [2]. The AviTag extends from residues 1–29, and protein A from residues 30–90; residues 1–38 are disordered in solution. Incorporation of protonated methyl groups of Leu (C^δ1^H_3_ and C^δ2^H_3_) in a fully deuterated background was carried out as described previously by growing the bacteria in minimal D_2_O medium with ammonium chloride as the sole nitrogen source, U-[^2^H]D-glucose as the main carbon source, and the appropriate α-keto acid precursor for ^13^CH_3_ labeling: α-ketoisovaleric acid (^13^C5, 98%, 3-D1, 98%) for Leu and Val (Cambridge Isotope Laboratories CDLM-4418-PK). Note there are no valines in AviTag-Protein A [2]. Nitroxide (R1 or R1p) spin labeling was carried out with S-(1-oxyl-2,2,5,5-tetramethyl-2,5-dihydro-1H-pyrrol-3-yl)methyl methanesulfonothioate (MTSL; Toronto Research Chemicals) or 2,5-Dihydro-2,2,5,5-tetraMethyl-3- [[(Methylsulfonyl)thio]Methyl]-4-(3 -pyridinyl)-1H-pyrrol-1-yloxy (R1p; Toronto Research Chemicals) as described previously [2]. Nitroxide labeling was verified by electrospray ionization mass spectrometry. The sample for EPR comprised 50 µM AviTag-Protein A, 0.85 mM KH_2_PO_4_, 25 mM Na_2_HPO_4_, pH 7.4, 75 mM NaCl, and d8-glycerol(30% v/v)/D_2_O (70% v/v).

### Pulsed Q-Band EPR Spectroscopy

Pulsed EPR data were collected at Q-band (33.8 GHz) and 50 K on a Bruker E-580 spectrometer equipped with a 300 W traveling-wave tube amplifier, a model ER5107D2 resonator, and a cryofree cooling unit, as described previously. DEER experiments were acquired using a conventional four-pulse sequence [1]. The observer and ELDOR pump pulses were separated by ca. 90 MHz with the observer π/2 and π pulses set to 12 and 24 ns, respectively, and the ELDOR π pulse to 10 ns. The pump frequency was centered at the Q-band nitroxide spectrum located at + 40 MHz from the center of the resonator frequency. The *τ*_1_ value of 400 ns for the first echo-period time was incremented eight times in 16 ns steps to average ^2^H modulation; the position of the ELDOR pump pulse was incremented in steps of Δ*t* = 8 ns. The bandwidth of the overcoupled resonator was 120 MHz. All DEER echo curves were acquired for *τ*_max_ = 4 μs to avoid the persistent “2 + 1” echo perturbation of the DEER echo curves at a time of about *τ*_1_ from the final observed *π* pulse. DEER data were recorded with values of the dipolar evolution time 2*τ*_2_ ranging from 10 to 40 μs for [Leu-C^δ^H_3_/^2^H] AviTag-Protein A. Individual isotope-labeled variants were immediately measured by EPR upon mixing with deuteratexd glycerol, upon which time delays and temperatures were applied. Between temperature adjustments, a dwell time of 2 h was applied to the instrument prior to continued measurement. Measurement times were approximately as follows: for 2*τ*_2_ = 10, 20 µs, 1–4 h; 30, 40 µs, 12–48 h. The measurement time largely varied due to increased signal-to-noise with increasing the temperature; the final signal-to-noise ratio was determined via DeerLab’s deerlab.der_snr module during global fitting [25]. The individual DEER time traces will be deposited on Figshare upon publication.

### Quantitative Analysis of a *T*_*m*_-Edited DEER Echo Curve Series

Analysis of a series of *T*_*m*_-edited DEER echo curves recorded over a range of τ_2_ values, especially when the P(r) distribution comprises several distance peaks (due to heterogeneous conformation states of the spin labels), requires a global fitting procedure in which all the DEER echo curves at the different *τ*_2_ values are fitted simultaneously using a shared set of Gaussians in which the peak positions and widths (at half-maximum), the relaxation times *T*_*m*_ are treated as global optimized parameters. Further, additional global optimized parameters describing the actual methyl rotation include the activation energy and its distribution. Trace-dependent local parameters include the decay rate constants for the background exponential function and the modulation depth. Here, we used a two-Gaussian distribution for fitting the DEER data, while this underfit a number of dipolar evolution time traces it was reasoned that based on the Tikhonov regularization results by DeerLab, the main contributors to the DEER signal were a Gaussian at 32 and 38 Å; additionally other Gaussian components would to low populated to isolate trustworthy information. It is important to point out that obtaining *P(r)* distributions from individual DEER echo curves using Tikhonov regularization implemented in DeerLab is regarded as the gold standard, therefore validated Tikhonov regularization was performed with the bootstrap analysis for uncertainty quantification via the bootan function in the DeerLab library, with the number of bootstrap samples evaluated set to 1000 [25]. Further, the regularization parameter was selected via the aicc criteria and an exponential background function was applied.

## Supplementary Information

Below is the link to the electronic supplementary material.

Supplementary file1 (DOCX 16964 KB)

## Data Availability

No datasets were generated or analysed during the current study.
