# Supplementary material for: Temperature-Dependent Rotation of Protonated Methyl Groups in Otherwise Deuterated Proteins Modulates DEER Distance Distributions
Source: Appl Magn Reson. 2024 Oct 3;56(1-2):91–102. doi: 10.1007/s00723-024-01720-5 (PMC11775055; doi:10.1007/s00723-024-01720-5)
Supplement: Supplementary file 1 — Supplementary file1 (DOCX 16964 KB) [file 723_2024_1720_MOESM1_ESM.docx]

**SUPPORTING INFORMATION**

**Temperature dependent rotation of protonated methyl groups in otherwise deuterated proteins modulates DEER distance distributions**

Thomas Schmidt*, Valentyn Stadnytskyi

Laboratory of Chemical Physics, National Institute of Diabetes and Digestive and Kidney Diseases, National Institutes of Health, Bethesda, MD 20892-0520, U.S.A.

**Experimental Procedures**

**Description of methyl rotation.** The activation energy landscape for a methyl label is given by equ S1 (Fig 1B), where N is the number of points in the activation energy grid each described by its index i, <E_a_> is the mean activation energy and σ is its distribution. This activation energy distribution is weighted by equ S2 giving a Gaussian probability density function, f_i_. The three-site jump constant for the individual conformers is obtained by the Arrhenius law (equ S3) where k_0_, R and T denote the three-site jump rate, ideal gas constant and temperature, which was validated for methyl groups for leucine or R1 system with limited conformational heterogeneity.[[4,5]](https://paperpile.com/c/IU5B3N/GyoE+kZwg) The subsequent modulation factor, k_app_ (equ S4), is obtained by the summation of weighted exponentials for individual conformers at electron evolution time 2τ_2_ (Fig 1C).

[
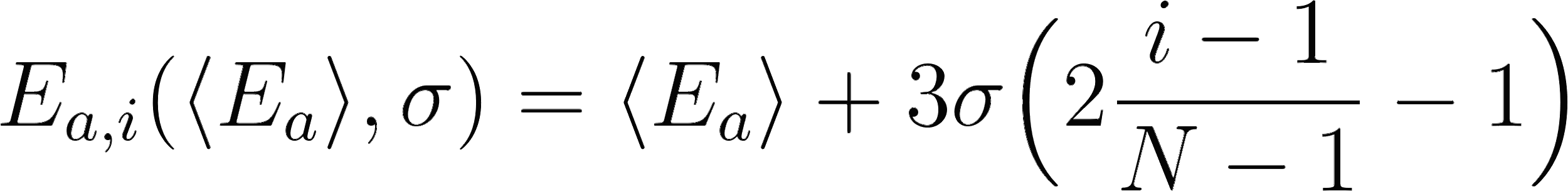
](https://latex-staging.easygenerator.com/eqneditor/editor.php?latex=E_%7Ba%2Ci%7D(%5Clangle%20E_%7Ba%7D%20%5Crangle%2C%5Csigma)%3D%26%20%5Clangle%20E_a%5Crangle%20%2B%203%5Csigma%20%5CBig(%202%5Cfrac%7Bi-1%7D%7BN-1%7D-1%5CBig)#0) (S1)

[
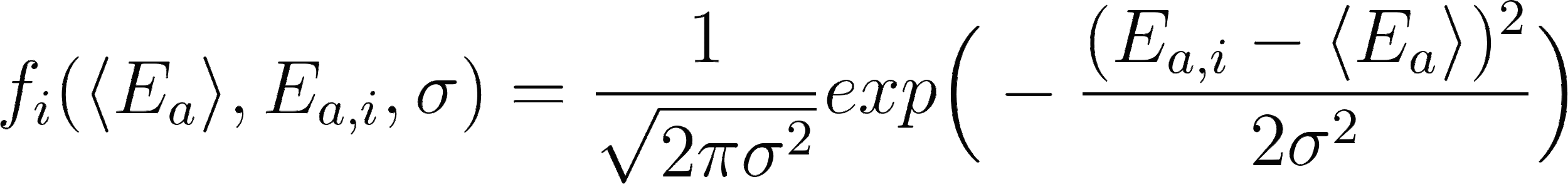
](https://latex-staging.easygenerator.com/eqneditor/editor.php?latex=f_i(%5Clangle%20E_%7Ba%7D%20%5Crangle%2CE_%7Ba%2Ci%7D%2C%5Csigma)%3D%20%26%20%5Cfrac%7B1%7D%7B%5Csqrt%7B2%5Cpi%5Csigma%5E2%7D%7D%20exp%5CBig(-%5Cfrac%7B(E_%7Ba%2Ci%7D-%5Clangle%20E_%7Ba%7D%5Crangle)%5E2%7D%7B2%5Csigma%5E2%7D%5CBig)#0) (S2)

[
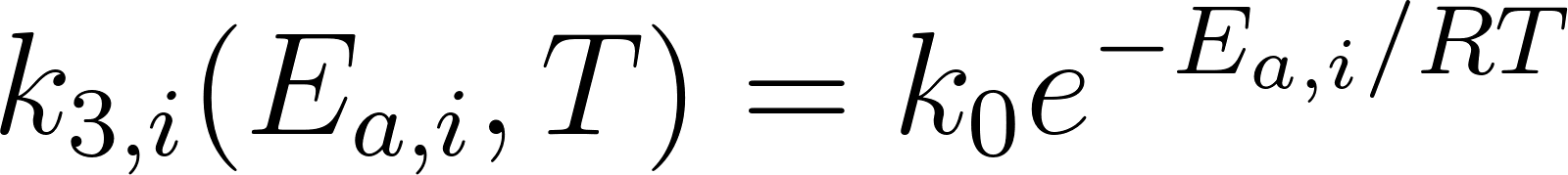
](https://latex-staging.easygenerator.com/eqneditor/editor.php?latex=k_%7B3%2Ci%7D(E_%7Ba%2Ci%7D%2CT)%3D%26%20%20%20k_0e%5E%7B-E_%7Ba%2Ci%7D%2F%7BRT%7D%7D#0) (S3)

[
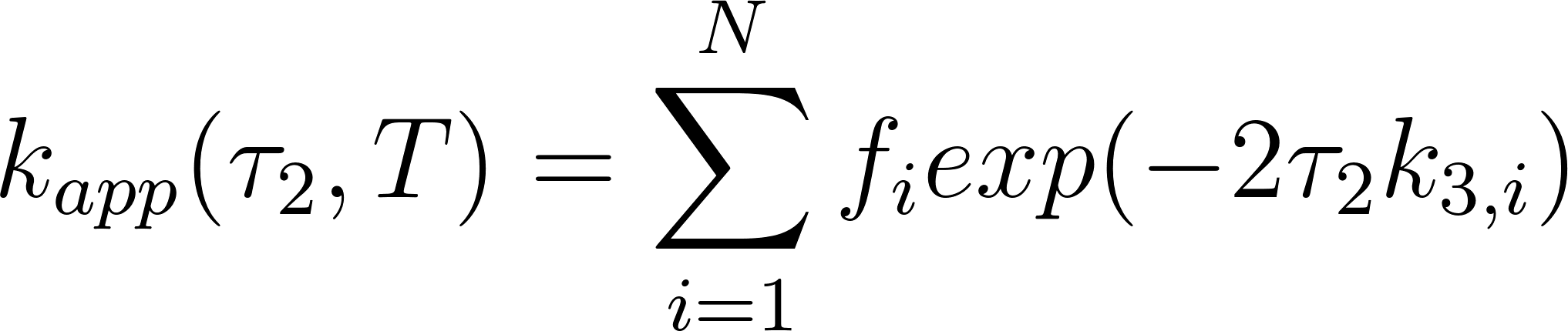
](https://latex-staging.easygenerator.com/eqneditor/editor.php?latex=k_%7Bapp%7D(%5Ctau_2%2C%20T)%3D%26%5Csum_%7Bi%3D1%7D%5E%7BN%7D%20f_i%20exp(-2%5Ctau_2k_%7B3%2Ci%7D)#0) (S4)

**Table S1** Isotope labeling schemes and their fitting values for two-Gaussian fit.

| protein A | MTSL | R (Å) | | σ (Å) | |
| --- | --- | --- | --- | --- | --- |
|  |  | R1 | R2 | σ1 | σ2 |
| *Distance and its distribution are restrained* | | | | | |
| ^2^H | ^2^H | 31.8±0.1 | 38.2±0.1 | 2.4±0.1 | 2.8±0.1 |
| ^2^H | ^1^H | 31.8±0.1 | 38.2±0.1 | 2.4±0.1 | 2.8±0.1 |
| ^1^H-Leu-CH_3_/^2^H | ^2^H | 31.9±0.1 | 38.7±0.1 | 2.5±0.1 | 2.7±0.1 |
| ^1^H-Leu-CH_3_/^2^H | ^1^H | 31.8±0.1 | 38.6±0.1 | 2.3±0.1 | 2.9±0.1 |
| *Distance, distribution,〈E_a_〉and* σ_a_  *are restrained* | | | | | |
| ^2^H | ^2^H | - | - | - | - |
| ^2^H | ^1^H | 31.9±0.1 | 38.4±0.1 | 2.3±0.1 | 2.8±0.1 |
| ^1^H-Leu-CH_3_/^2^H | ^2^H | 32.1±0.1 | 39.1±0.1 | 2.2±0.1 | 2.9±0.1 |
| ^1^H-Leu-CH_3_/^2^H | ^1^H | 31.9±0.1 | 38.4±0.1 | 2.2±0.1 | 2.8±0.1 |

**Table S2** Isotope labeling schemes and their fitting values for two-Gaussian fit.

| protein A | R1p | R (Å) | | σ (Å) | | X^2^ |
| --- | --- | --- | --- | --- | --- | --- |
|  |  | R1 | R2 | σ1 | σ2 |  |
| ^2^H | ^1^H | 31.8±0.1 | 38.3±0.1 | 1.4±0.1 | 2.6±0.1 | 1.1^#^/1.2* |
| ^1^H-Leu-CH_3_/^2^H | ^1^H | 32.5±0.1 | 38.5±0.1 | 1.6±0.1 | 2.1±0.1 | 1.0^#^/1.1* |

# Averages for model-free fits to the individual DEER echo curves using DeerLab with validated Tikhonov regularization (n=1000).

* Gaussian global fits based on the program DD/GLADDvu, incorporated into an inhouse Python program to the complete set of Q-band DEER echo curves recorded over a series of temperate and τ_2_ values . For the presented two-Gaussian global fit, only the mean distances and corresponding widths are constrained to be invariant.


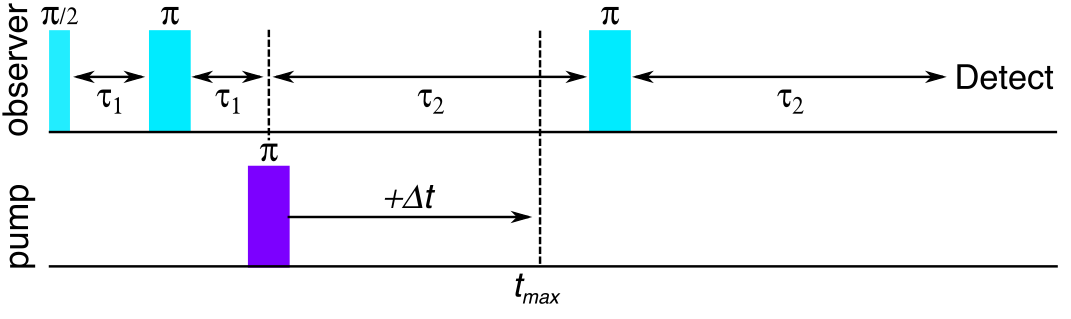


**Figure S1.** Pulse schemes for four-pulse DEER experiments[[3]](https://paperpile.com/c/IU5B3N/6295). In the current work, the duration of the blue π/2 and π pulses are 12 and 24 ns, respectively, and that of the purple π pulse is 10 ns. For all presented experiments the т_2_ was kept fixed at 150 ns and the Δt was kept at 8 ns. The values of т_2_ alternated between 5, 10, 15 and 20 μsn while the t_max_ remained constant at 4 μs.


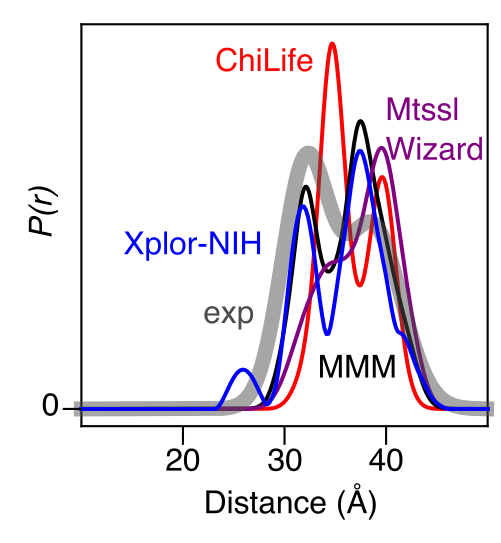


**Figure S2.** Predicted distance distributions between the nitroxide spin labels at Q39C-R1 and K88C-R1 in AviTag-Protein A confirms a bimodal distribution. The spin label conformer ensembles were calculated from the coordinates of Protein A (PDB code 1BDD) using the rotamer library program MMM (black)[[3,6]](https://paperpile.com/c/IU5B3N/6295+caRR), MtsslWizard (lilac)[[7]](https://paperpile.com/c/IU5B3N/cZod), ChiLife (red)[[8]](https://paperpile.com/c/IU5B3N/Rbxk) and Xplor-NIH (blue)[[9,10]](https://paperpile.com/c/IU5B3N/qyEV+JBVW) in comparison to the experimental distribution (gray). Q39C-R1 exists in two distinct ensemble populations a and b (see Fig. 1A of main text). The distances are calculated to the carbon atoms of the methyl groups and displayed as the integral of the *P*(*r*) distributions. While the linker length of R1 and R1p are the same no predictions for R1p were attempted as the addition of the pyridyl group applies spacial restrictions that require further investigation.


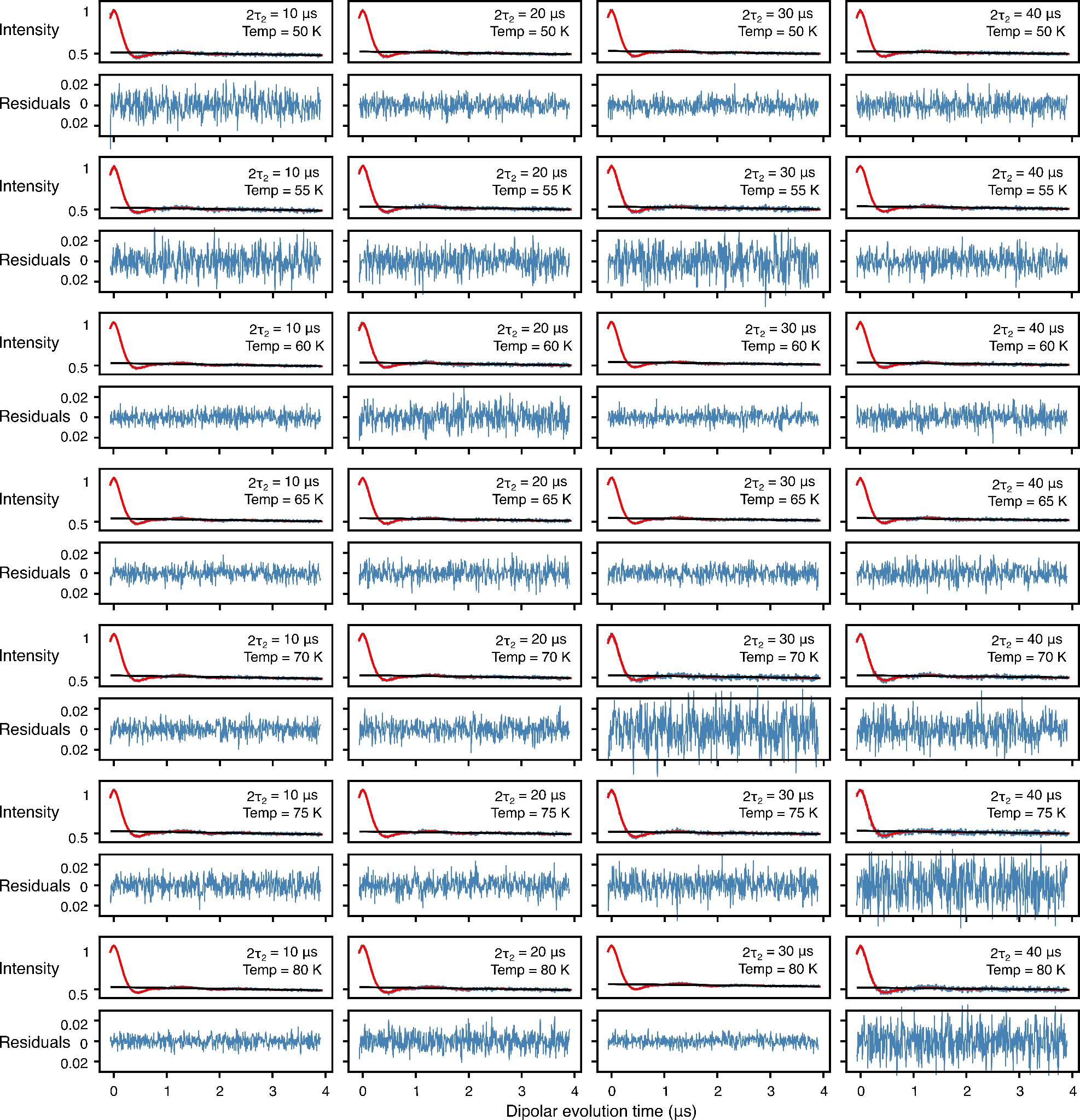


**Figure S3.** Analysis of Q-band DEER data acquired for spin-labeled protein A (Q39C-R1/K88C-R1), the protein as well as the MTSL label were fully deuterated (^2^H-Leu, ^2^H-R1), by using validated Tikhonov regularization (n=1000). In each panel, the top half displays the experimental (blue) and bestfit (red) DEER echo curves; the bottom half shows the corresponding residuals between experimental and calculated curves. The data at each temperature and 2т_2_ combination were fitted individually using validated Tikhonov regulation in the program DeerLab.[[11]](https://paperpile.com/c/IU5B3N/aZSx) The mean normalized χ^2^ value of the fits is given in Table 1 of the main text.


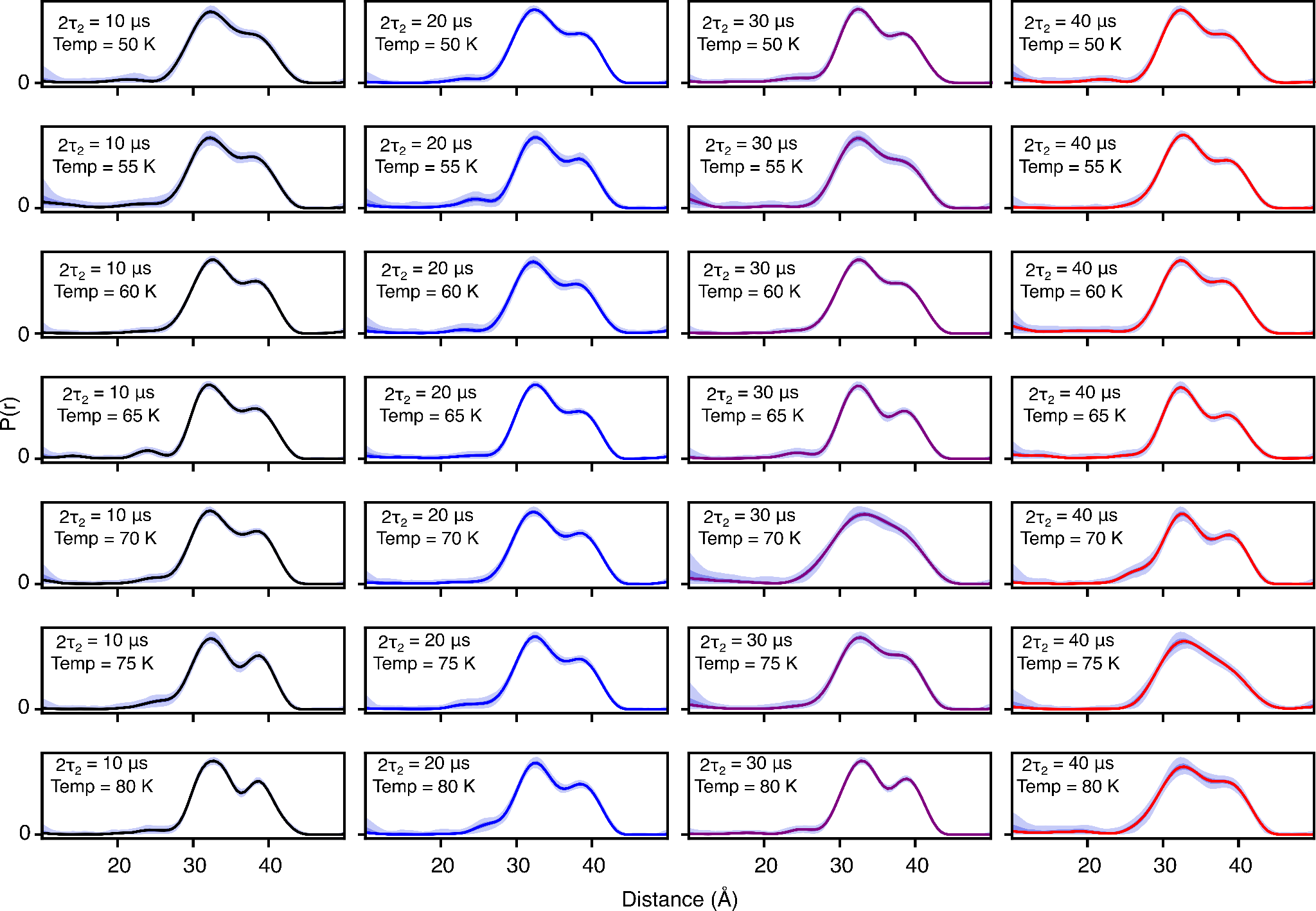


**Figure S4.** DEER-derived *P(r)* distributions for spin-labeled protein A (Q39C-R1/K88C-R1) by using validated Tikhonov regularization (n=1000)[[11]](https://paperpile.com/c/IU5B3N/aZSx), the protein as well as the MTSL label were fully deuterated (^2^H-Leu, ^2^H-R1). The fits to the experimental DEER echo curves are shown in Fig. S3. The solid lines present 2т_2_ of 10 (*black*), 20 (*blue*), 30 (*lilac*) and 40 μs (*red*). The shaded region below presents the 95% (*light violet*) and 50% (*dark violet*) confidence interval.


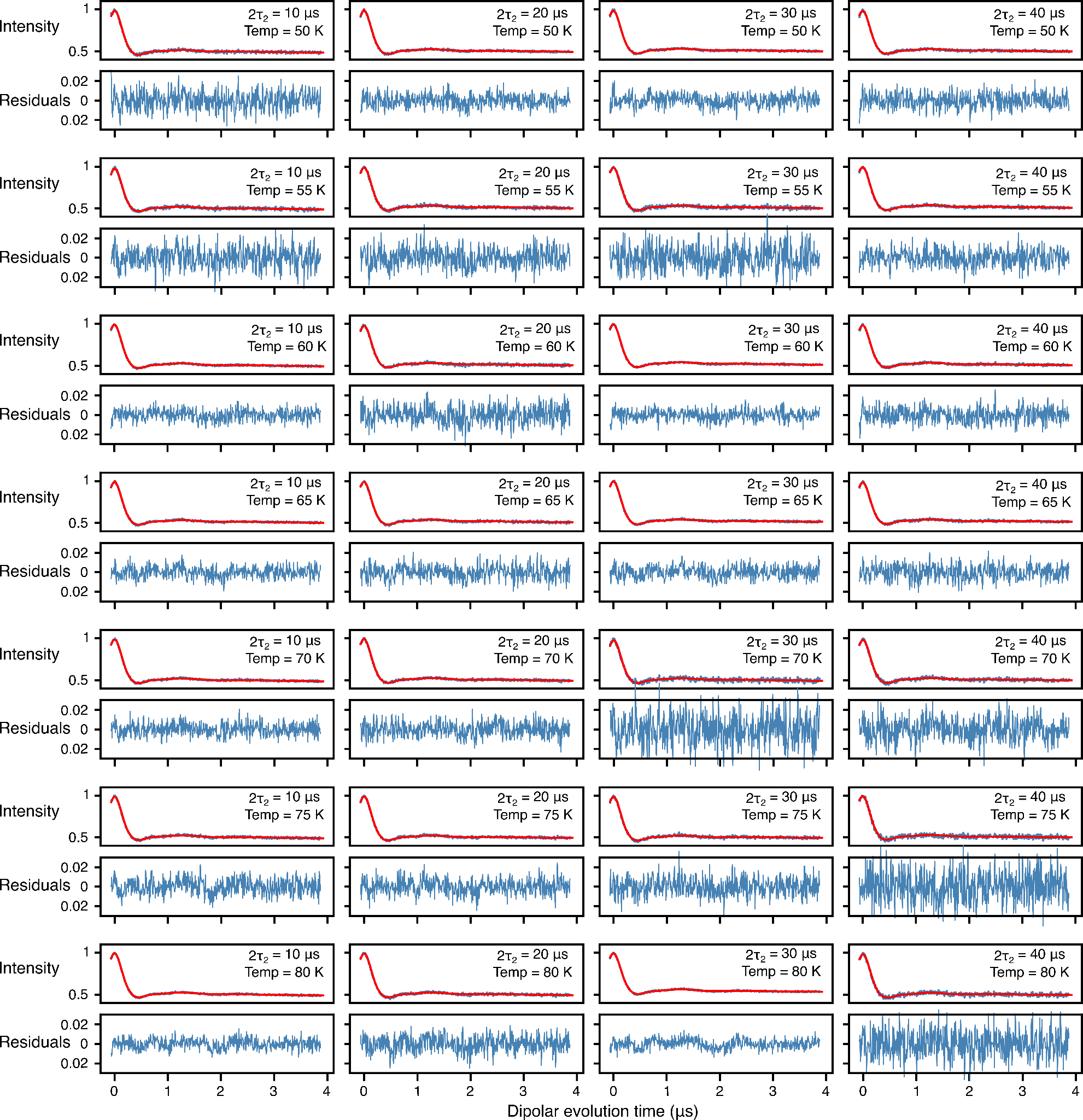


**Figure S5.** Global analysis of Q-band DEER data acquired for spin-labeled protein A (Q39C-R1/K88C-R1), the protein as well as the MTSL label were fully deuterated (^2^H-Leu, ^2^H-R1), using a 2-Gaussian restrained fit. In each panel, the top half displays the experimental (blue) and bestfit (red) DEER echo curves; the bottom half shows the corresponding residuals between experimental and calculated curves. The data at each temperature and 2т_2_ combination were fitted simultaneously with the peak positions and corresponding peak widths in the P(r) distributions treated as global parameters using an in-house Python script [[9]](https://paperpile.com/c/IU5B3N/qyEV) based on the program DD/GLADDvu.[[12,13]](https://paperpile.com/c/IU5B3N/rCrQ+e6QU) The values of the reduced χ^2^ and optimized global parameters are provided in Table 1 of the main text.


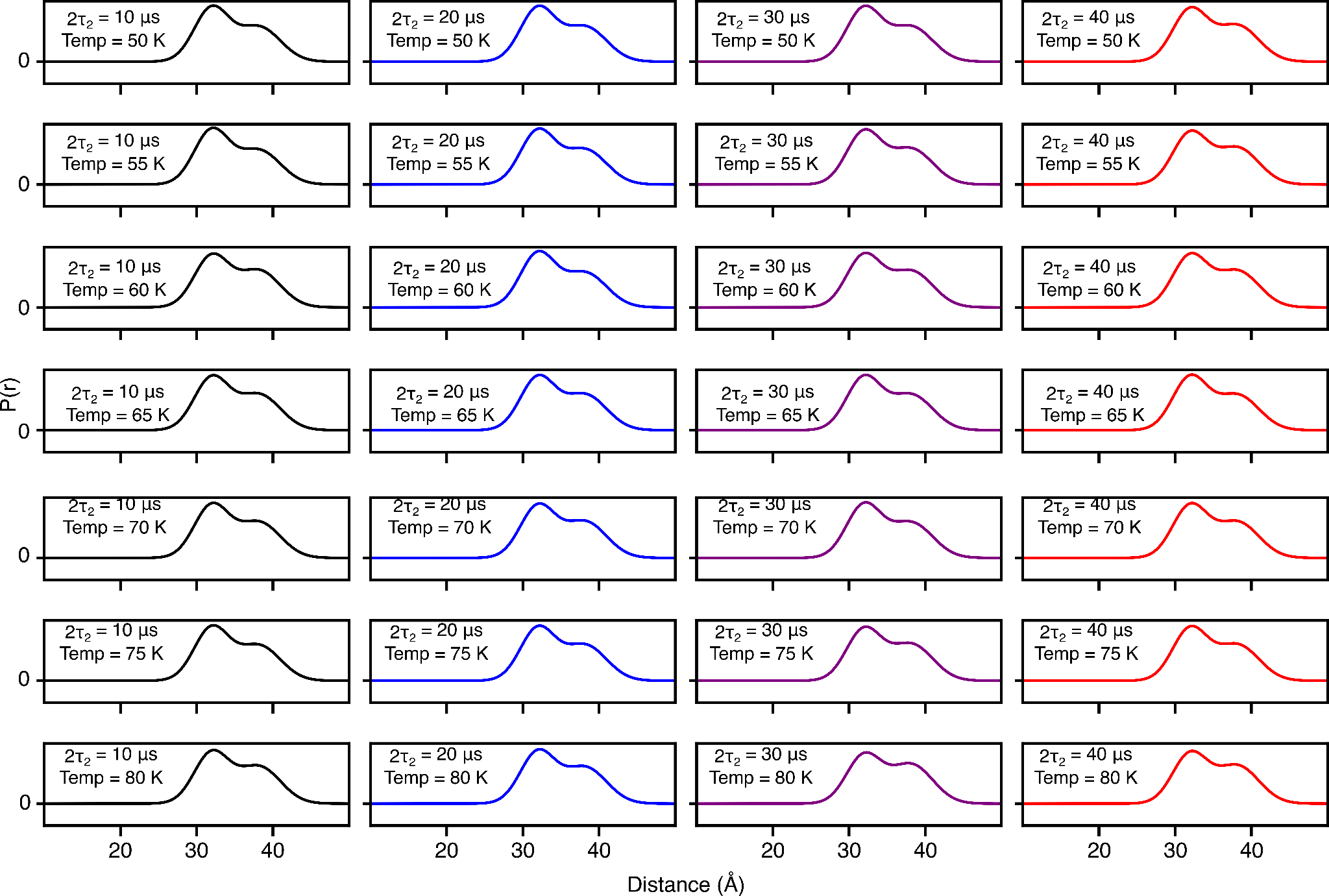


**Figure S6.** DEER-derived *P(r)* distributions for spin-labeled protein A (Q39C-R1/K88C-R1) by using two-Gaussian global fit in which the peak positions and corresponding widths are treated as global parameters (see main text for details).[[10]](https://paperpile.com/c/IU5B3N/JBVW) The protein as well as the MTSL label were fully deuterated (^2^H-Leu, ^2^H-R1). The fits to the experimental DEER echo curves are shown in Fig. S5. The solid lines present 2т_2_ of 10 (*black*), 20 (*blue*), 30 (*lilac*) and 40 μs (*red*).

**
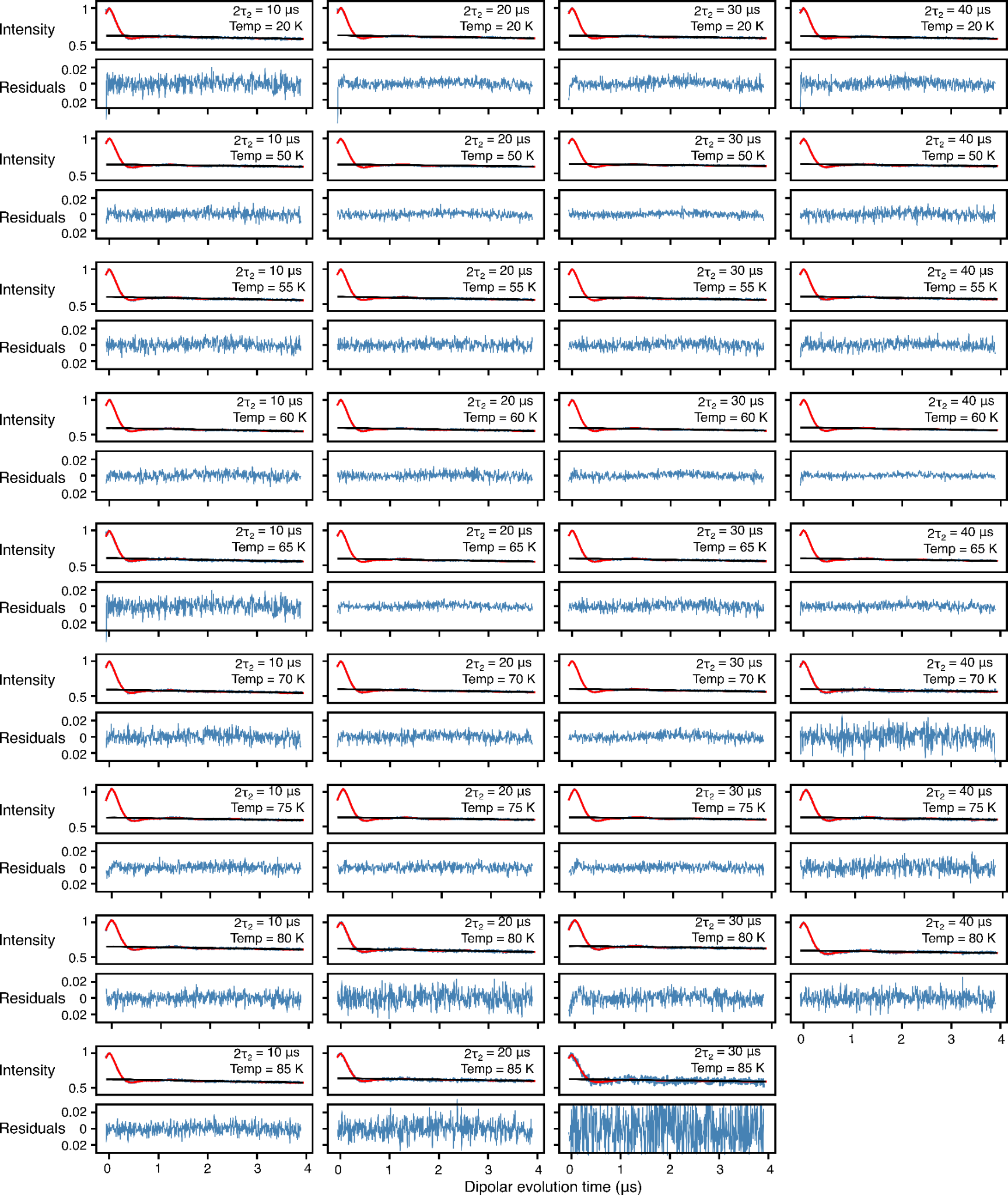
**

**FigureS7.** Analysis of Q-band DEER data acquired for spin-labeled protein A (Q39C-R1/K88C-R1), the protein was fully deuterated while the MTSL label was protonated (^2^H-Leu, ^1^H-R1), by using validated Tikhonov regularization (n=1000). In each panel, the top half displays the experimental (blue) and bestfit (red) DEER echo curves; the bottom half shows the corresponding residuals between experimental and calculated curves. The data at each temperature and 2т_2_ combination were fitted individually using validated Tikhonov regulation in the program DeerLab.[[11]](https://paperpile.com/c/IU5B3N/aZSx) The mean normalized χ^2^ value of the fits is given in Table 1 of the main text.

**
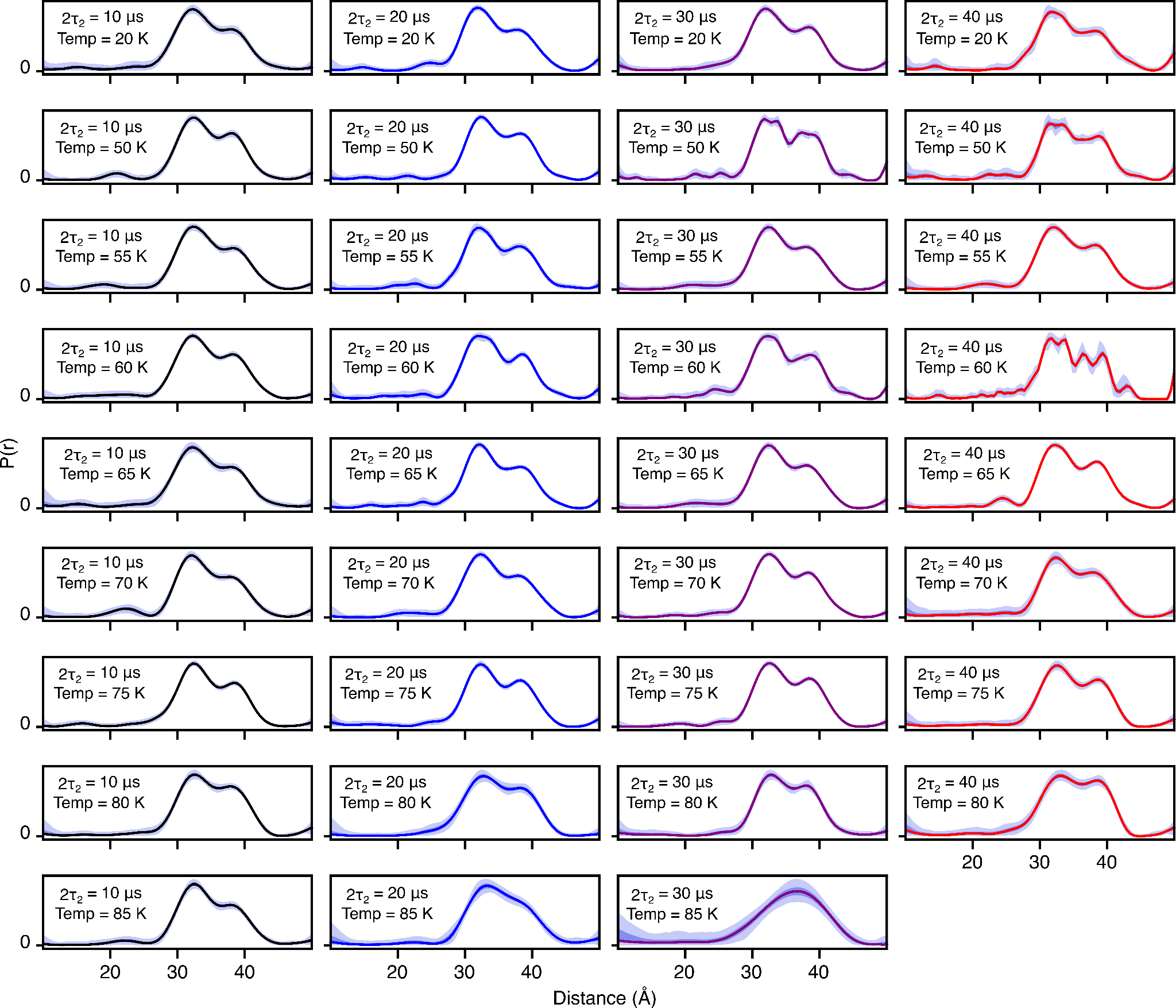
**

**Figure S8.** DEER-derived *P(r)* distributions for spin-labeled protein A (Q39C-R1/K88C-R1) by using validated Tikhonov regularization (n=1000), the protein was fully deuterated while the MTSL label was protonated (^2^H-Leu, ^1^H-R1). The fits to the experimental DEER echo curves are shown in Fig. S7. The solid lines present 2т_2_ of 10 (*black*), 20 (*blue*), 30 (*lilac*) and 40 μs (*red*). The shaded region below presents the 95% (*light violet*) and 50% (*dark violet*) confidence interval.

**
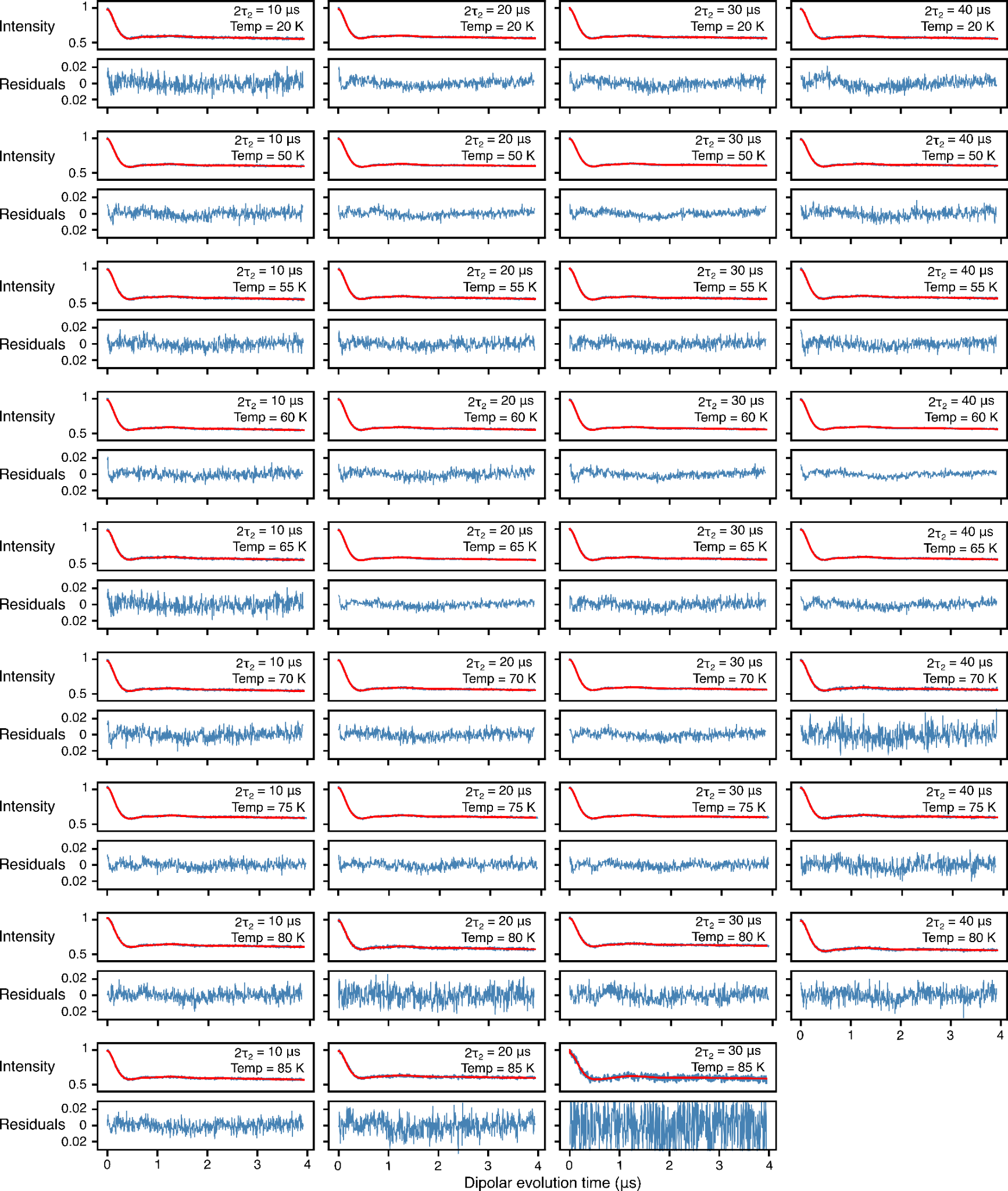
**

**Figure S9.** Global analysis of Q-band DEER data acquired for spin-labeled protein A (Q39C-R1/K88C-R1), the protein was fully deuterated while the MTSL label was protonated (^2^H-Leu, ^1^H-R1), using a 2-Gaussian restrained fit. In each panel, the top half displays the experimental (blue) and bestfit (red) DEER echo curves; the bottom half shows the corresponding residuals between experimental and calculated curves. The data at each temperature and 2т_2_ combination were fitted simultaneously with the peak positions and corresponding peak widths in the P(r) distributions treated as global parameters using an in-house Python script[[9,10]](https://paperpile.com/c/IU5B3N/qyEV+JBVW) based on the program DD/GLADDvu.[[12,13]](https://paperpile.com/c/IU5B3N/rCrQ+e6QU) The values of the reduced χ^2^ and optimized global parameters are provided in Table 1 of the main text.

**
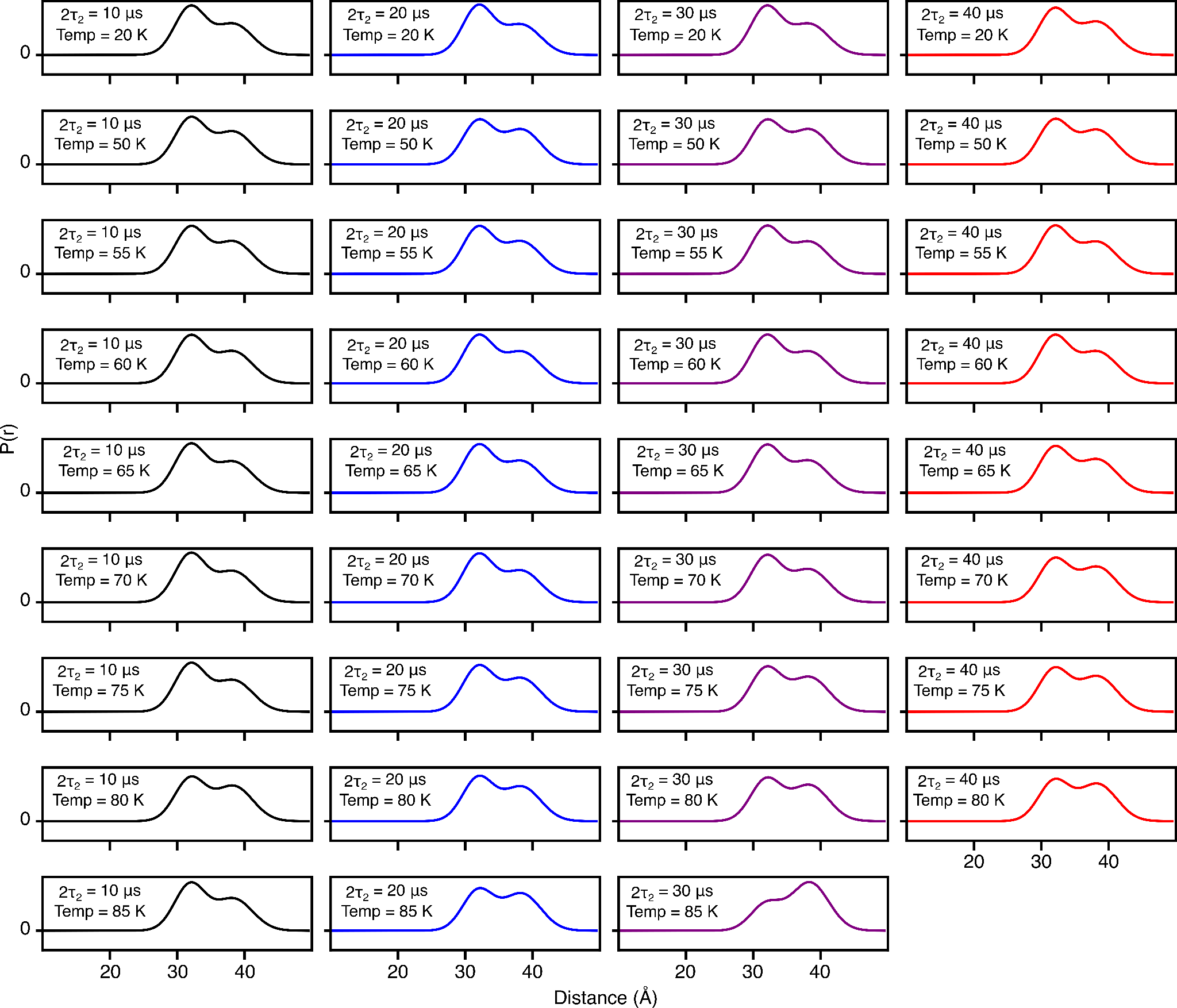
**

**Figure S10.** DEER-derived *P(r)* distributions for spin-labeled protein A (Q39C-R1/K88C-R1) by using two-Gaussian global fit in which the peak positions and corresponding widths are treated as global parameters (see main text for details). The protein was fully deuterated while the MTSL label was protonated (^2^H-Leu, ^1^H-R1). The fits to the experimental DEER echo curves are shown in Fig. S9. The solid lines present 2т_2_ of 10 (*black*), 20 (*blue*), 30 (*lilac*) and 40 μs (*red*).

**
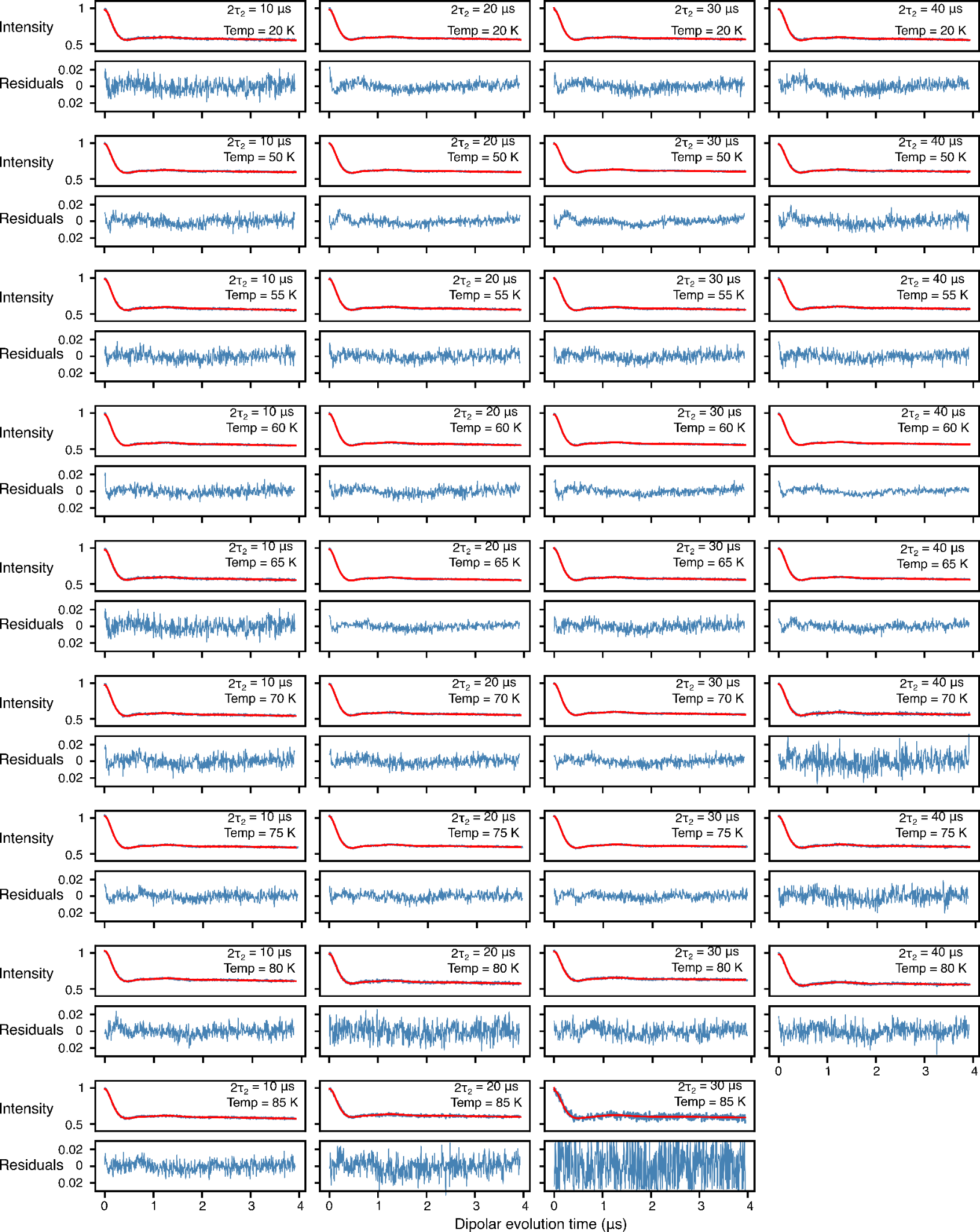
**

**Figure S11**. Global analysis of Q-band DEER data acquired for spin-labeled protein A (Q39C-R1/K88C-R1), the protein was fully deuterated while the MTSL label was protonated (^2^H-Leu, ^1^H-R1), using a 2-Gaussian restrained fit while Gaussian ratios were restrained by the methyl rotation as dictated by equ 5. In each panel, the top half displays the experimental (blue) and bestfit (red) DEER echo curves; the bottom half shows the corresponding residuals between experimental and calculated curves. The data at each temperature and 2т_2_ combination were fitted simultaneously, setting activation energy and its width, the peak positions and corresponding peak widths in the *P(r)* distributions as global parameters using an in-house Python script[[9]](https://paperpile.com/c/IU5B3N/qyEV) based on the program DD/GLADDvu.[[12,13]](https://paperpile.com/c/IU5B3N/rCrQ+e6QU) The values of the reduced χ^2^ and optimized global parameters are provided in Table 1 of the main text.

**
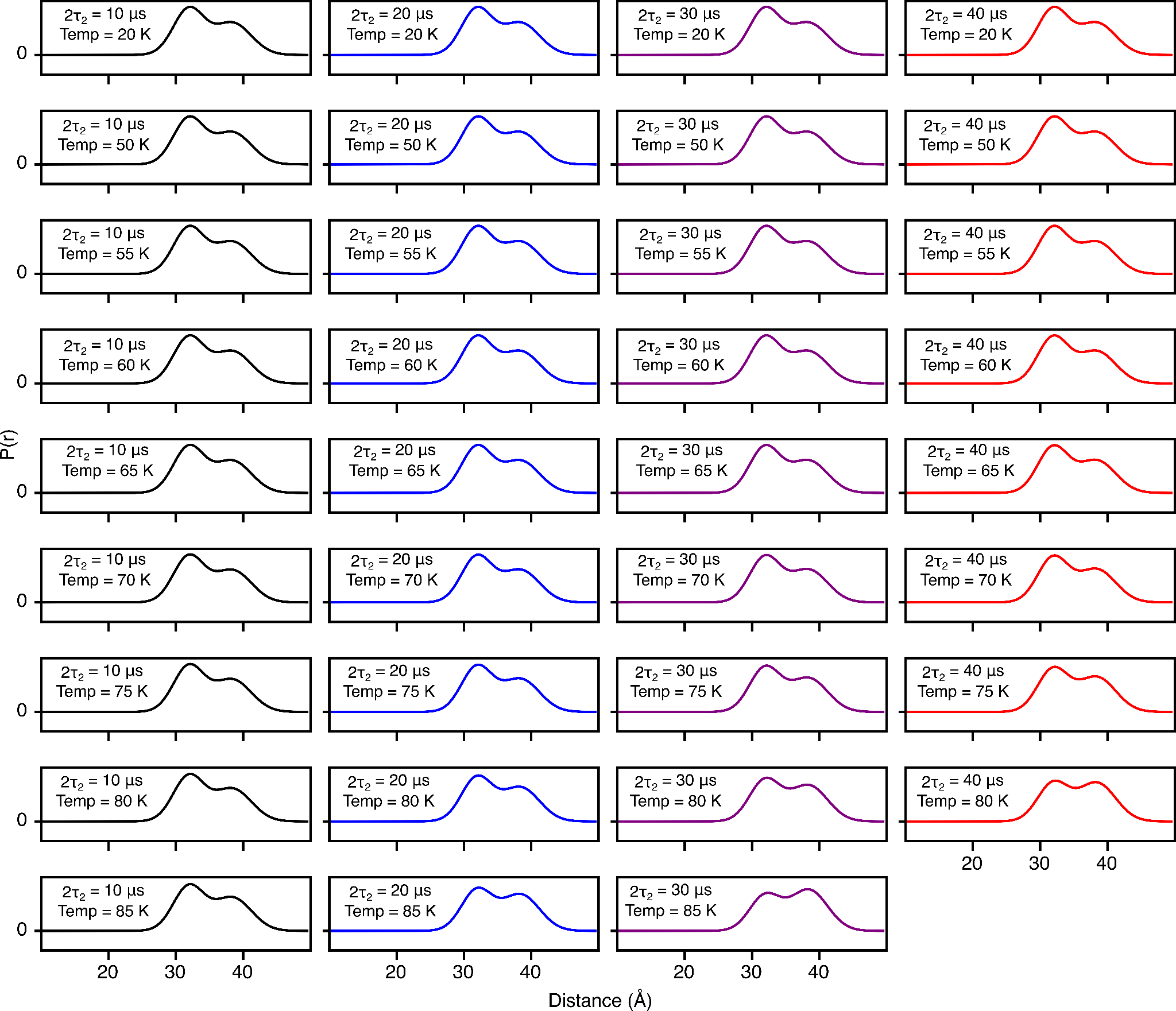
**

**Figure S12.** DEER-derived *P(r)* distributions for spin-labeled protein A (Q39C-R1/K88C-R1) by using two-Gaussian global fit in which the peak positions and corresponding widths are treated as global parameters. Further, the Gaussian ratios are dictated by the methyl rotation, therefore the activation energy and its width were treated as global parameters (see main text for details). The protein was fully deuterated while the MTSL label was protonated (^2^H-Leu, ^1^H-R1). The fits to the experimental DEER echo curves are shown in Fig. S11. The solid lines present 2т_2_ of 10 (*black*), 20 (*blue*), 30 (*lilac*) and 40 μs (*red*). The shaded region below presents the 95% (*light violet*) and 50% (*dark violet*) confidence interval.

**
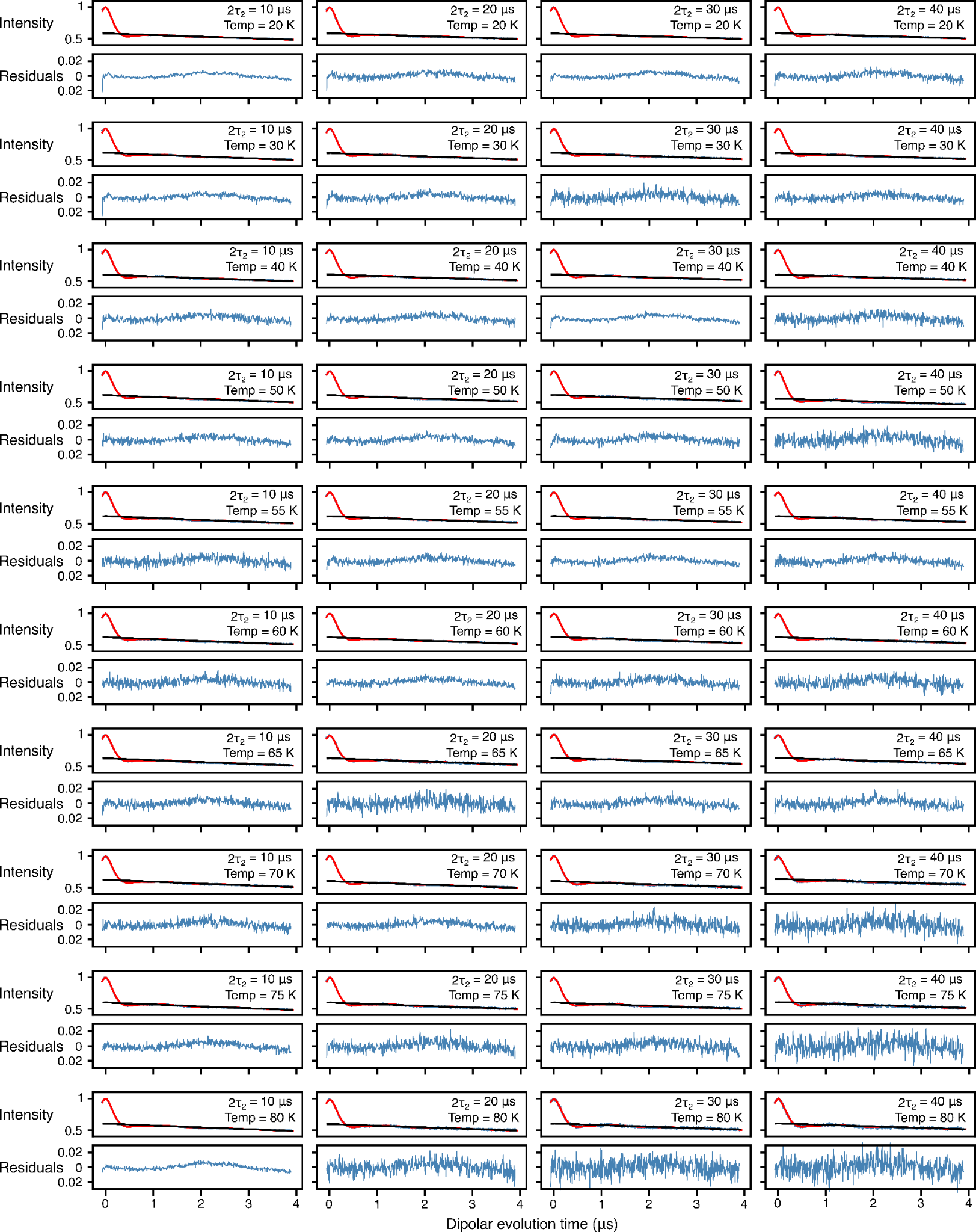
**

**FigureS13.** Analysis of Q-band DEER data acquired for spin-labeled protein A (Q39C-R1/K88C-R1), the protein was fully deuterated with site specific leucine protonation while the MTSL label was deuterated (^1^H-Leu, ^2^H-R1), by using validated Tikhonov regularization (n=1000). In each panel, the top half displays the experimental (blue) and bestfit (red) DEER echo curves; the bottom half shows the corresponding residuals between experimental and calculated curves. The data at each temperature and 2т_2_ combination were fitted individually using validated Tikhonov regulation in the program DeerLab.[[11]](https://paperpile.com/c/IU5B3N/aZSx) The mean normalized χ^2^ value of the fits is given in Table 1 of the main text.

**
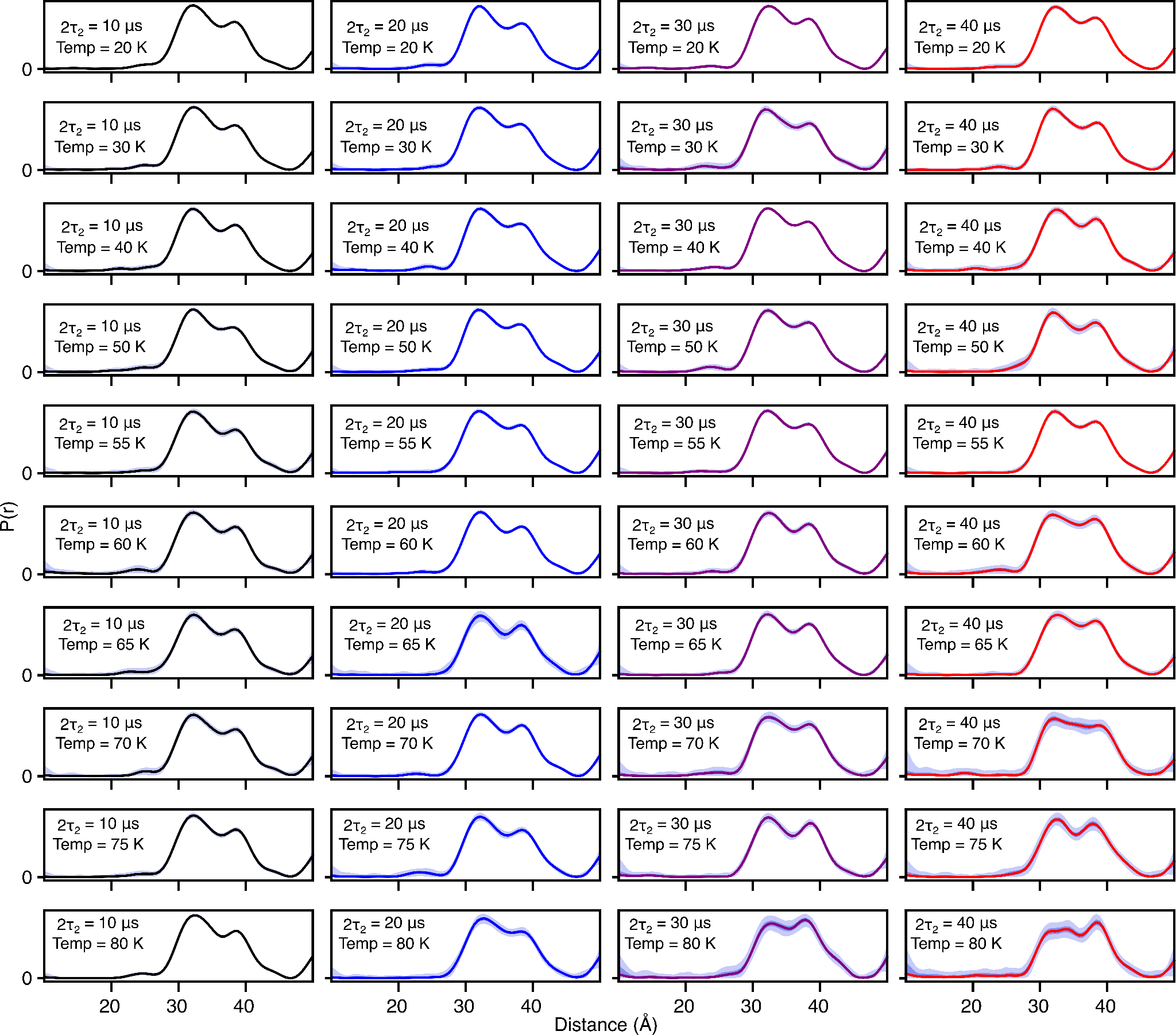
**

**Figure S14.** DEER-derived *P(r)* distributions for spin-labeled protein A (Q39C-R1/K88C-R1) by using validated Tikhonov regularization (n=1000), the protein was fully deuterated while the leucine sidechains were protonated (^1^H-Leu, ^2^H-R1). The fits to the experimental DEER echo curves are shown in Fig. S13. The solid lines present 2т_2_ of 10 (*black*), 20 (*blue*), 30 (*lilac*) and 40 μs (*red*). The shaded region below presents the 95% (*light violet*) and 50% (*dark violet*) confidence interval.

**
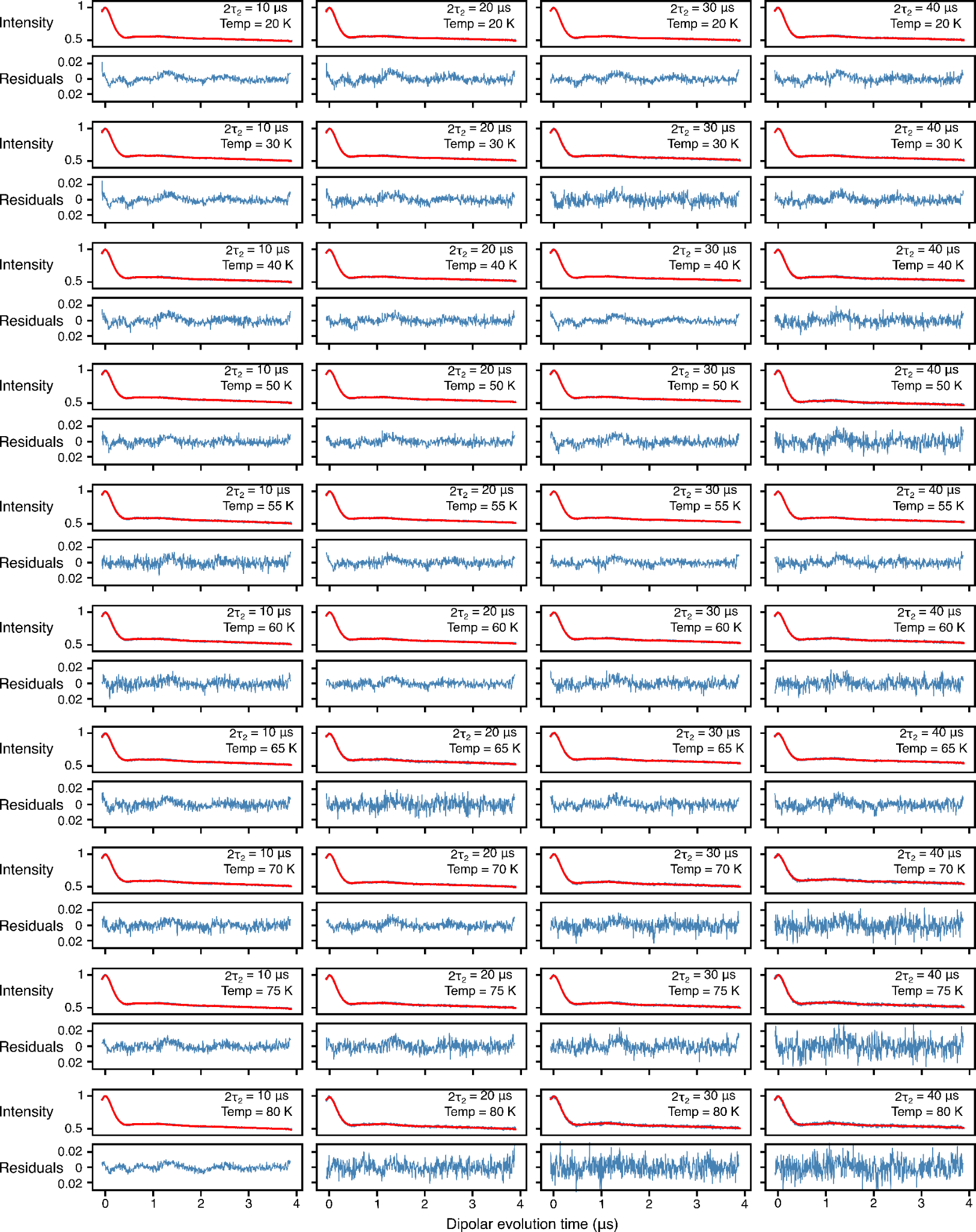
**

**Figure S15.** Global analysis of Q-band DEER data acquired for spin-labeled protein A (Q39C-R1/K88C-R1), the protein was fully deuterated with site specific leucine protonation while the MTSL label was deuterated (^1^H-Leu, ^2^H-R1), using a 2-Gaussian restrained fit. In each panel, the top half displays the experimental (blue) and bestfit (red) DEER echo curves; the bottom half shows the corresponding residuals between experimental and calculated curves. The data at each temperature and 2т_2_ combination were fitted simultaneously with the peak positions and corresponding peak widths in the P(r) distributions treated as global parameters using an in-house Python script[[9,10]](https://paperpile.com/c/IU5B3N/qyEV+JBVW) based on the program DD/GLADDvu.[[12,13]](https://paperpile.com/c/IU5B3N/rCrQ+e6QU) The values of the reduced χ^2^ and optimized global parameters are provided in Table 1 of the main text.

**
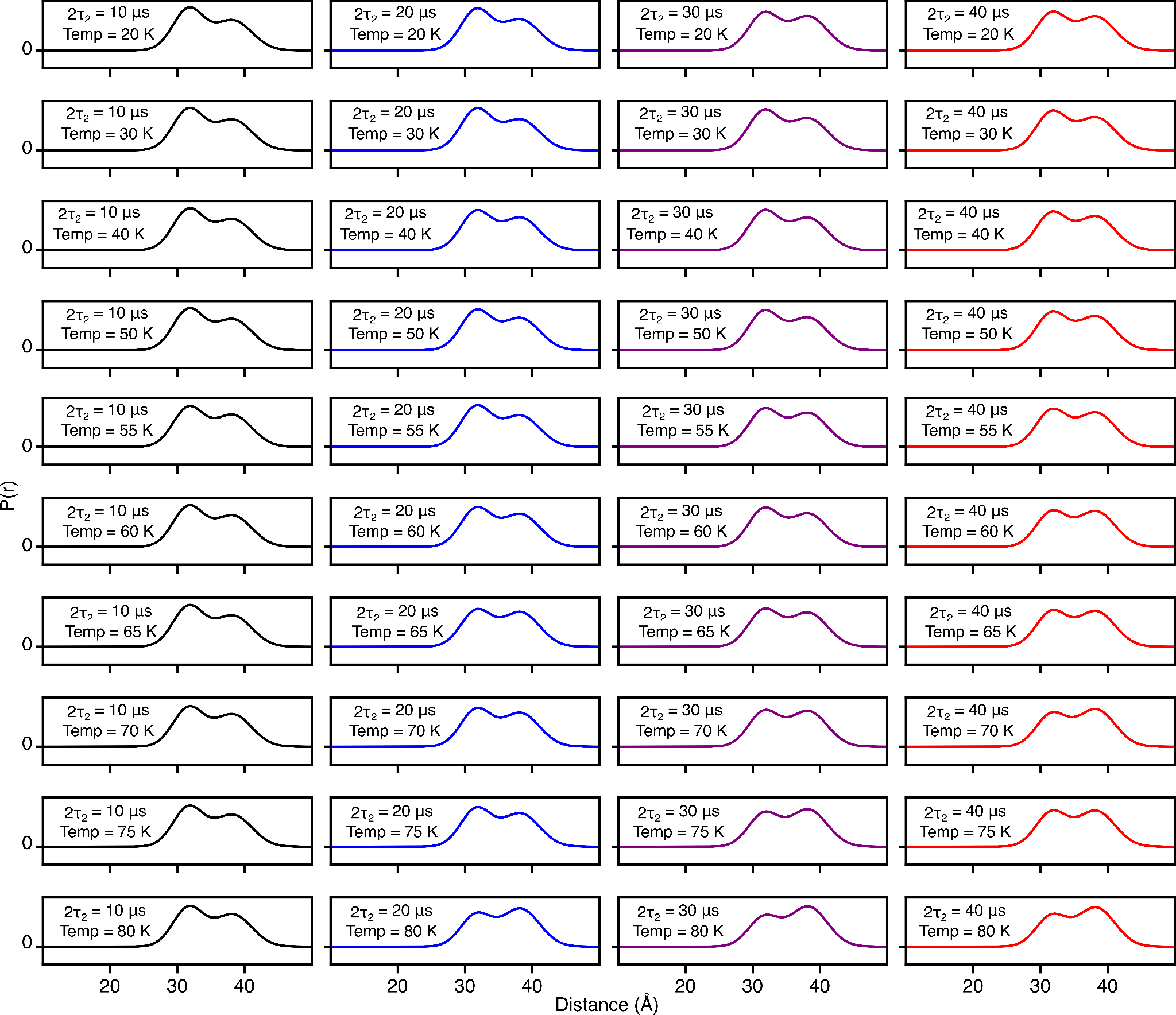
**

**Figure S16.** DEER-derived *P(r)* distributions for spin-labeled protein A (Q39C-R1/K88C-R1) by using two-Gaussian global fit in which the peak positions and corresponding widths are treated as global parameters (see main text for details). The protein was fully deuterated while the leucine side chains were protonated (^1^H-Leu, ^2^H-R1). The fits to the experimental DEER echo curves are shown in Fig. S15. The solid lines present 2т_2_ of 10 (*black*), 20 (*blue*), 30 (*lilac*) and 40 μs (*red*).

**
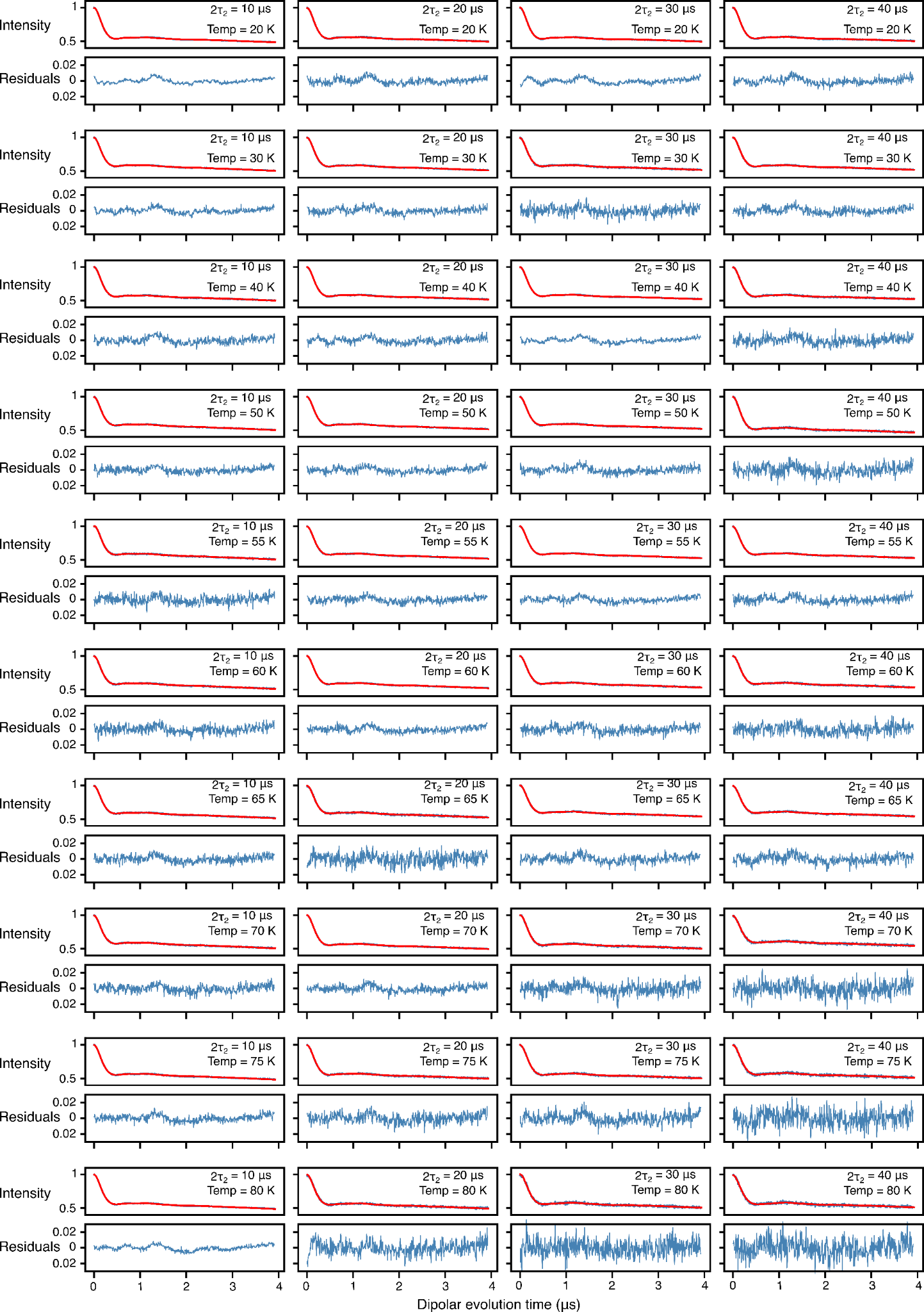
**

**Figure S17.** Global analysis of Q-band DEER data acquired for spin-labeled protein A (Q39C-R1/K88C-R1), the protein was fully deuterated with site specific leucine protonation while the MTSL label was deuterated (^1^H-Leu, ^2^H-R1), using a 2-Gaussian restrained fit while Gaussian ratios were restrained by the methyl rotation as dictated by equ 5. In each panel, the top half displays the experimental (blue) and bestfit (red) DEER echo curves; the bottom half shows the corresponding residuals between experimental and calculated curves. The data at each temperature and 2т_2_ combination were fitted simultaneously, setting activation energy and its width, the peak positions and corresponding peak widths in the P(r) distributions as global parameters using an in-house Python script[[9]](https://paperpile.com/c/IU5B3N/qyEV) based on the program DD/GLADDvu.[[12,13]](https://paperpile.com/c/IU5B3N/rCrQ+e6QU) The values of the reduced χ^2^ and optimized global parameters are provided in Table 1 of the main text.

**
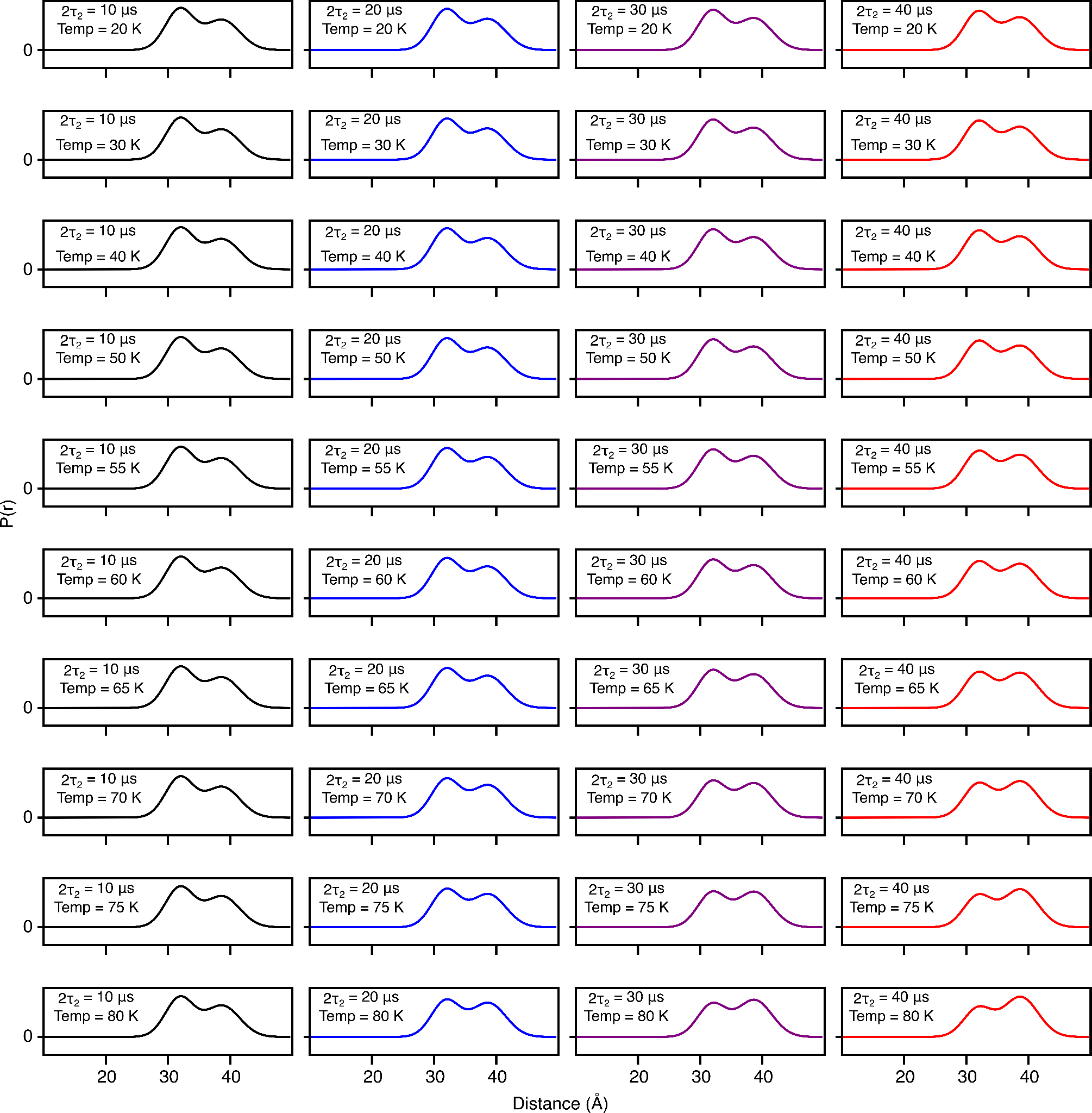
**

**Figure S18** DEER-derived *P(r)* distributions for spin-labeled protein A (Q39C-R1/K88C-R1) by using two-Gaussian global fit in which the peak positions and corresponding widths are treated as global parameters. Further, the Gaussian ratios are dictated by the methyl rotation, therefore the activation energy and its width were treated as global parameters (see main text for details). The protein was fully deuterated while the leucine side chain was protonated (^1^H-Leu, ^2^H-R1). The fits to the experimental DEER echo curves are shown in Fig. S17. The solid lines present 2т_2_ of 10 (*black*), 20 (*blue*), 30 (*lilac*) and 40 μs (*red*). The shaded region below presents the 95% (*light violet*) and 50% (*dark violet*) confidence interval.

**
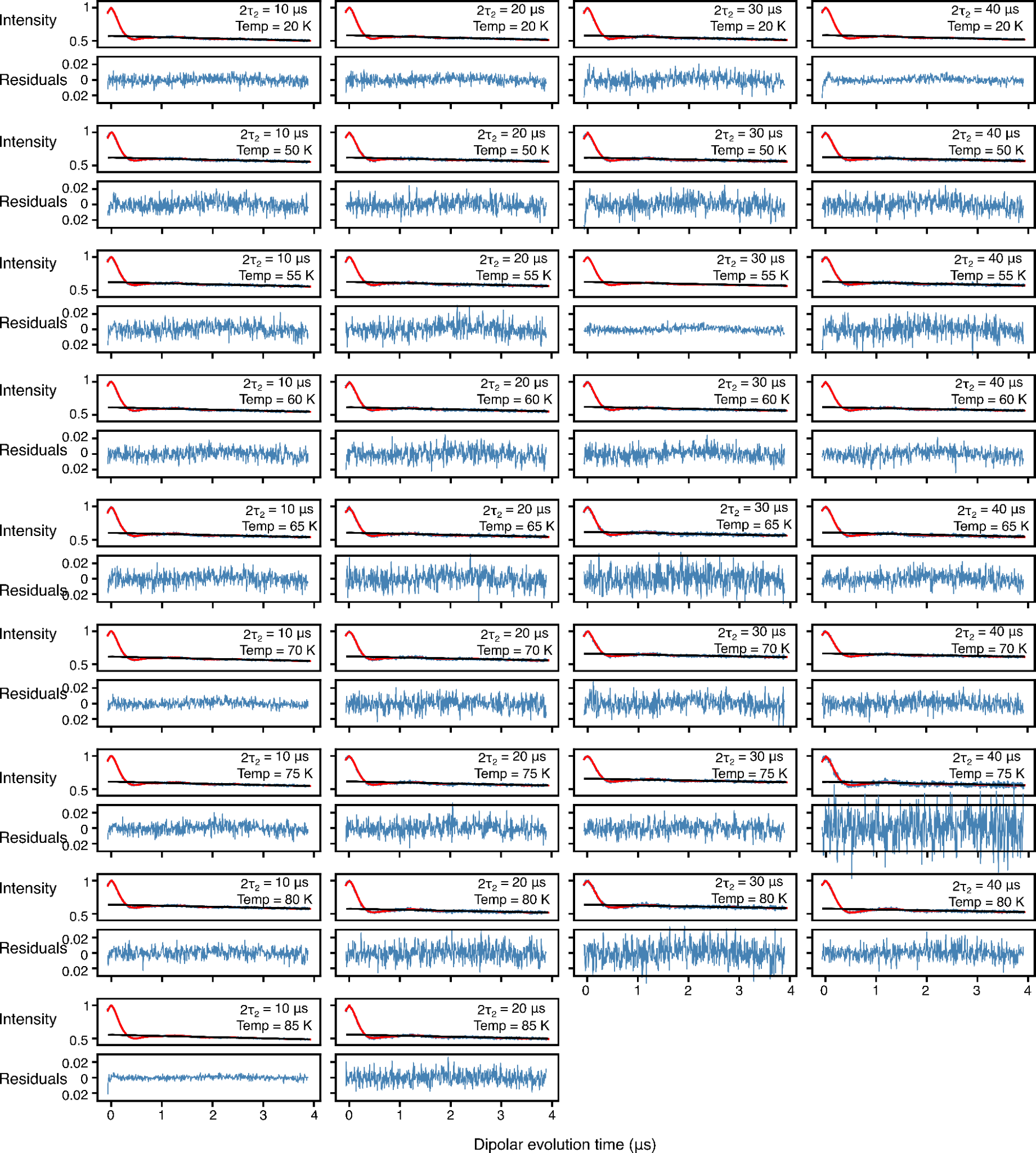
**

**FigureS19.** Analysis of Q-band DEER data acquired for spin-labeled protein A (Q39C-R1/K88C-R1), the protein was fully deuterated with site specific leucine protonation while the MTSL label was protonated (^1^H-Leu, ^1^H-R1), by using validated Tikhonov regularization (n=1000). In each panel, the top half displays the experimental (blue) and bestfit (red) DEER echo curves; the bottom half shows the corresponding residuals between experimental and calculated curves. The data at each temperature and 2т_2_ combination were fitted individually using validated Tikhonov regulation in the program DeerLab.[[11]](https://paperpile.com/c/IU5B3N/aZSx) The mean normalized χ^2^ value of the fits is given in Table 1 of the main text.

**
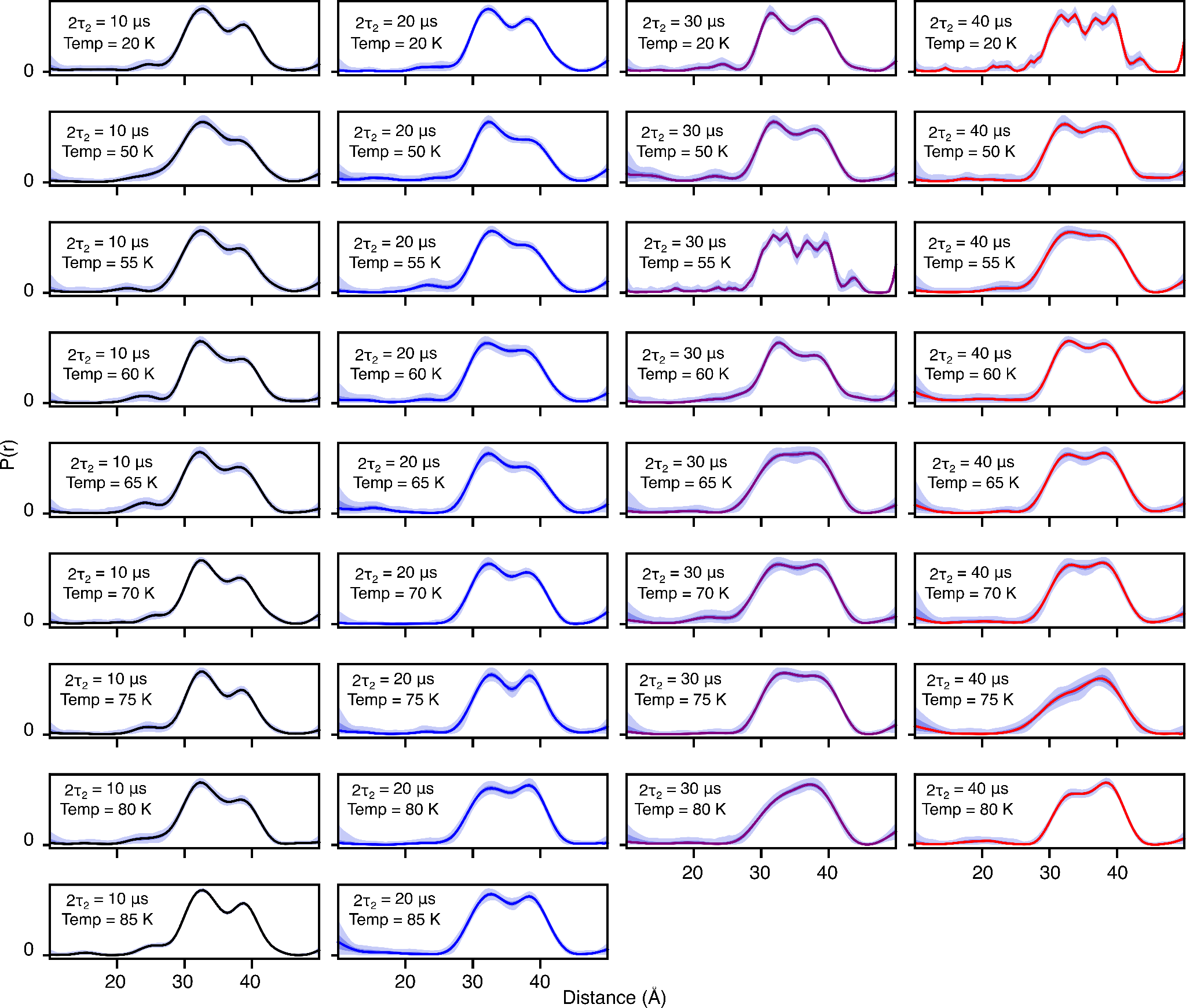
**

**Figure S20.** DEER-derived *P(r)* distributions for spin-labeled protein A (Q39C-R1/K88C-R1) by using validated Tikhonov regularization (n=1000), the protein was fully deuterated while the MTSL label and the leucine sidechains were protonated (^1^H-Leu, ^1^H-R1). The fits to the experimental DEER echo curves are shown in Fig. S19. The solid lines present 2т_2_ of 10 (*black*), 20 (*blue*), 30 (*lilac*) and 40 μs (*red*). The shaded region below presents the 95% (*light violet*) and 50% (*dark violet*) confidence interval.

**
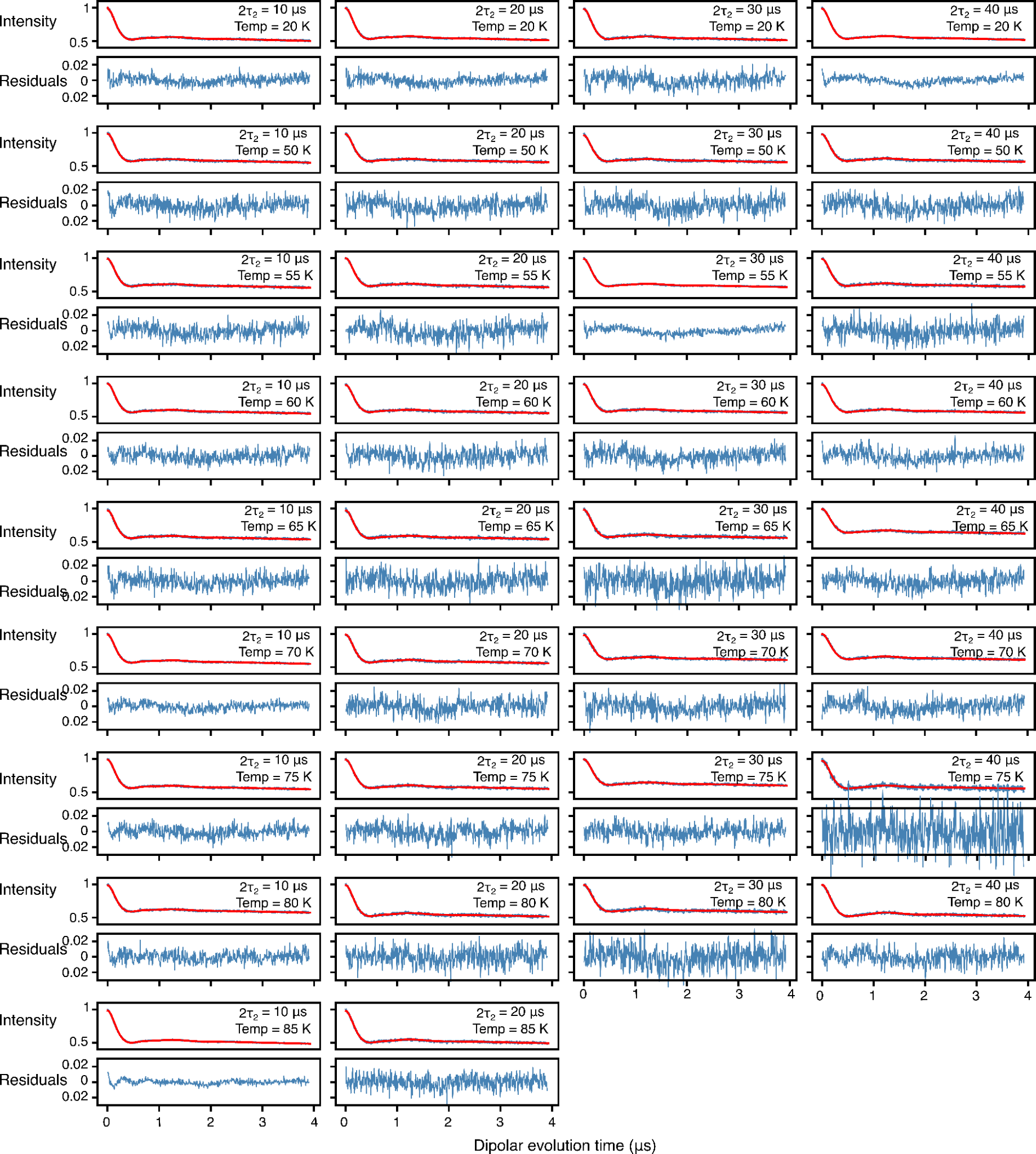
**

**Figure S21.** Global analysis of Q-band DEER data acquired for spin-labeled protein A (Q39C-R1/K88C-R1) using a 2-Gaussian restrained fit,the protein was fully deuterated with protonation of leucine sidechains and MTSL label (^1^H-Leu, ^1^H-R1), . In each panel, the top half displays the experimental (blue) and bestfit (red) DEER echo curves; the bottom half shows the corresponding residuals between experimental and calculated curves. The data at each temperature and 2т_2_ combination were fitted simultaneously with the peak positions and corresponding peak widths in the P(r) distributions treated as global parameters using an in-house Python script[[9,10]](https://paperpile.com/c/IU5B3N/qyEV+JBVW) based on the program DD/GLADDvu.[[11–13]](https://paperpile.com/c/IU5B3N/aZSx+rCrQ+e6QU) The values of the reduced χ^2^ and optimized global parameters are provided in Table 1 of the main text.

**
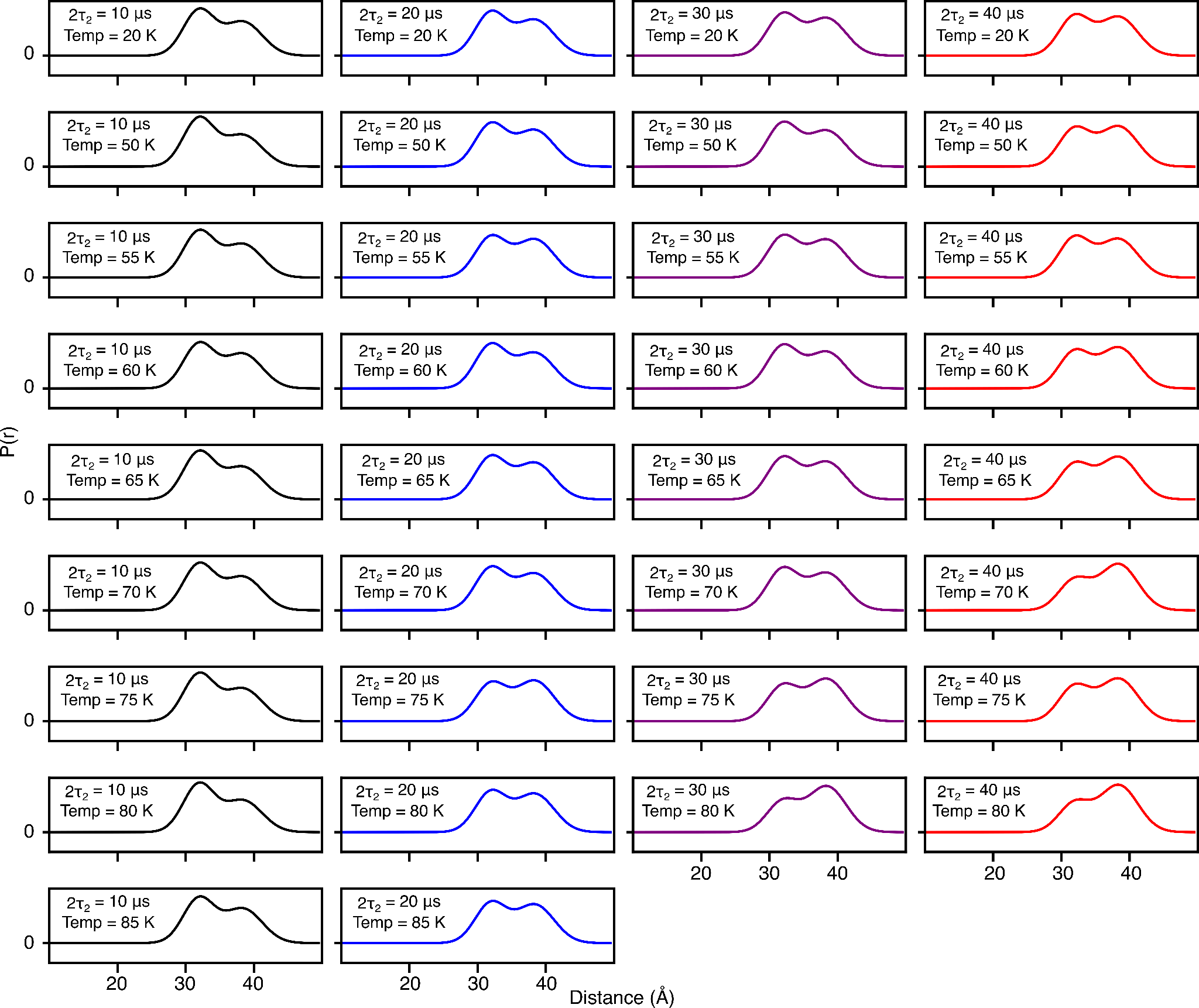
**

**Figure S22.** DEER-derived *P(r)* distributions for spin-labeled protein A (Q39C-R1/K88C-R1) by using two-Gaussian global fit in which the peak positions and corresponding widths are treated as global parameters (see main text for details). The protein was fully deuterated while the leucine side chains and MTSL label were protonated (^1^H-Leu, ^1^H-R1). The fits to the experimental DEER echo curves are shown in Fig. S21. The solid lines present 2т_2_ of 10 (*black*), 20 (*blue*), 30 (*lilac*) and 40 μs (*red*).

**
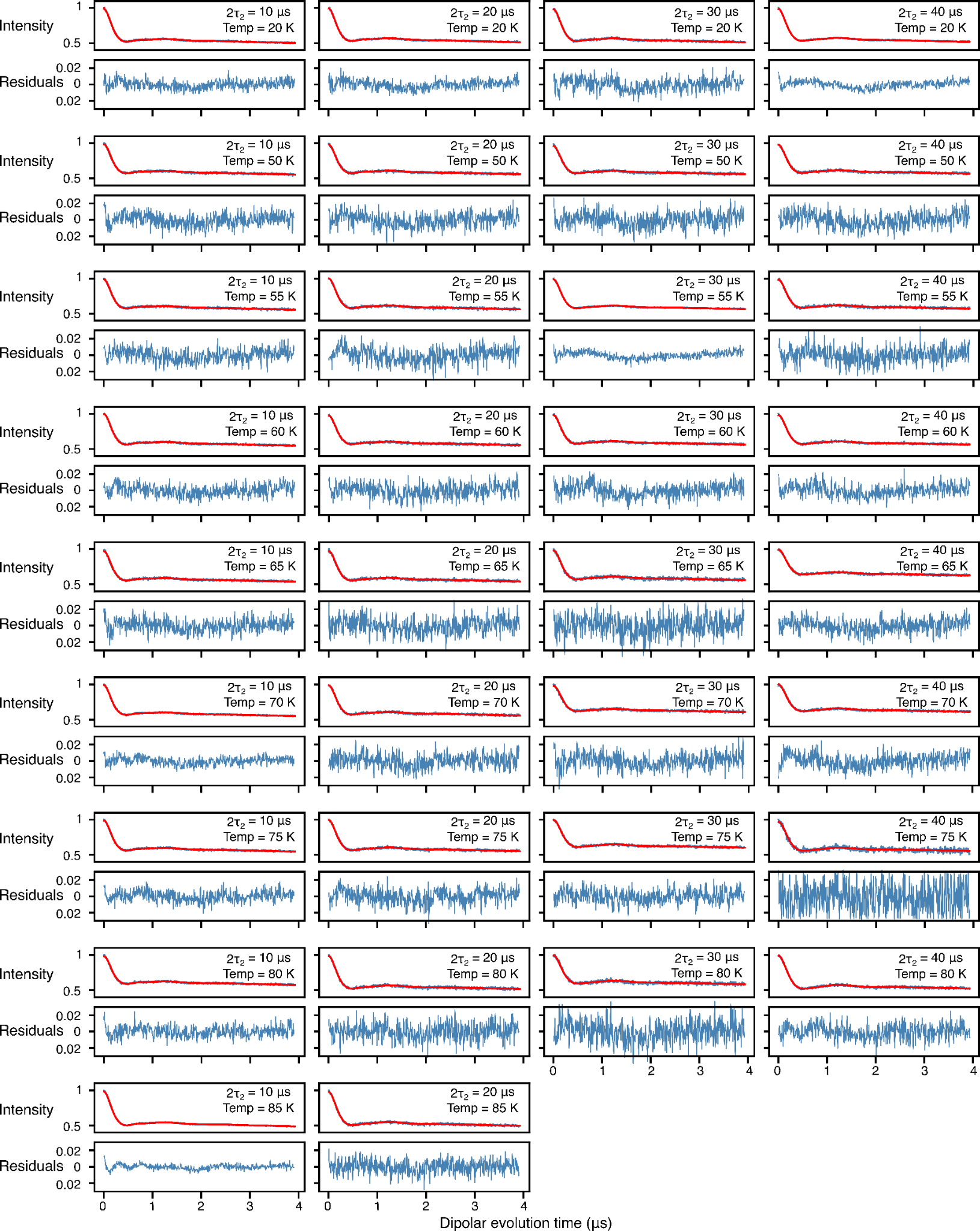
**

**Figure S23.** Global analysis of Q-band DEER data acquired for spin-labeled protein A (Q39C-R1/K88C-R1), the protein was fully deuterated with site specific leucine protonation and the MTSL label was protonated (^1^H-Leu, ^1^H-R1), using a 2-Gaussian restrained fit while Gaussian ratios were restrained by the methyl rotation as dictated by equ 5. In each panel, the top half displays the experimental (blue) and bestfit (red) DEER echo curves; the bottom half shows the corresponding residuals between experimental and calculated curves. The data at each temperature and 2т_2_ combination were fitted simultaneously, setting activation energy and its width, the peak positions and corresponding peak widths in the P(r) distributions as global parameters using an in-house Python script[[9]](https://paperpile.com/c/IU5B3N/qyEV) based on the program DD/GLADDvu.[[12,13]](https://paperpile.com/c/IU5B3N/rCrQ+e6QU) The values of the reduced χ^2^ and optimized global parameters are provided in Table 1 of the main text.

**
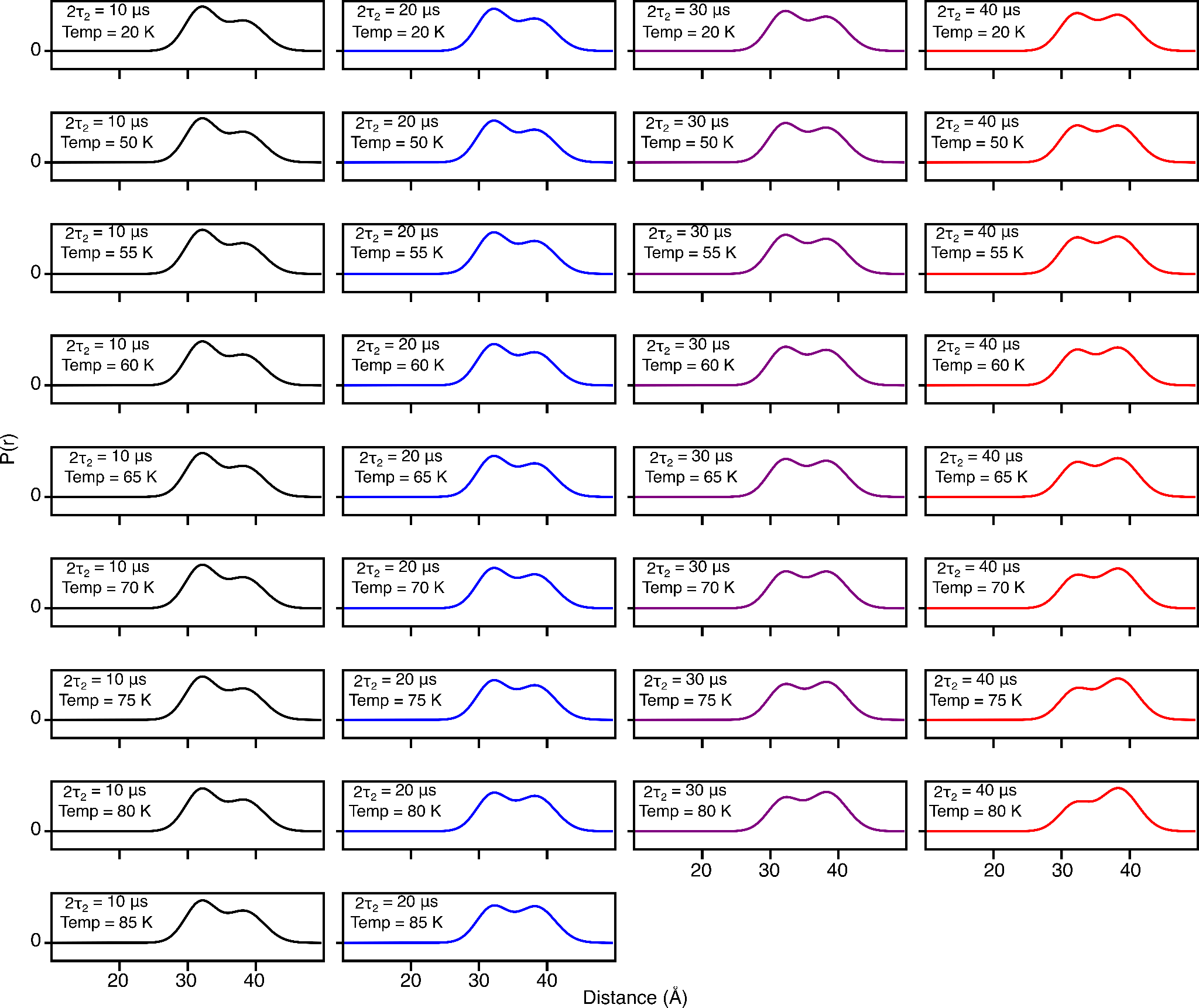
**

**Figure S24.** DEER-derived *P(r)* distributions for spin-labeled protein A (Q39C-R1/K88C-R1) by using two-Gaussian global fit in which the peak positions and corresponding widths are treated as global parameters. Further, the Gaussian ratios are dictated by the methyl rotation, therefore the activation energy and its width were treated as global parameters (see main text for details). The protein was fully deuterated while the leucine side chain and MTSL label were protonated (^1^H-Leu, ^1^H-R1). The fits to the experimental DEER echo curves are shown in Fig. S23. The solid lines present 2т_2_ of 10 (*black*), 20 (*blue*), 30 (*lilac*) and 40 μs (*red*). The shaded region below presents the 95% (*light violet*) and 50% (*dark violet*) confidence interval.

**
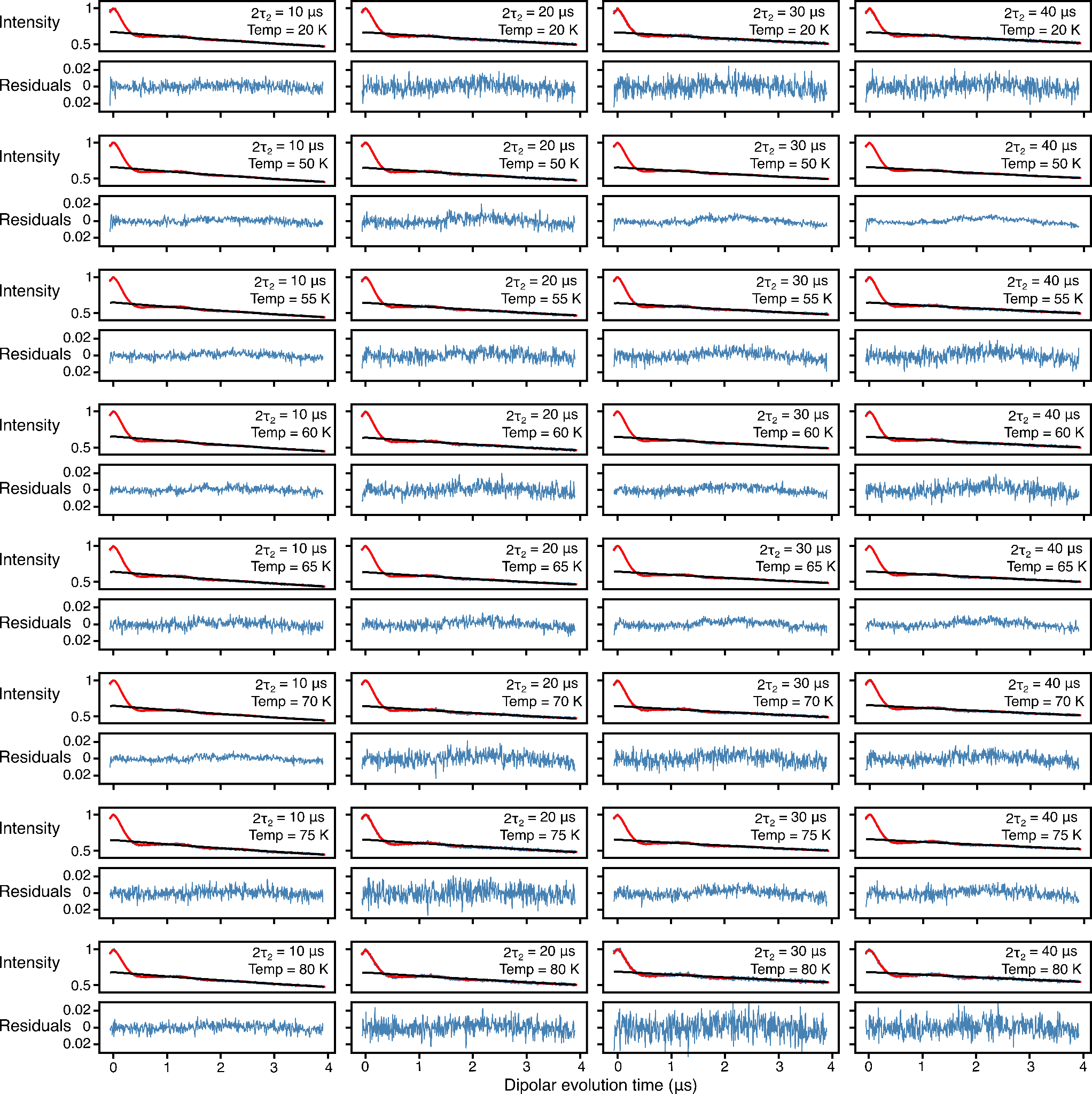
**

**FigureS25.** Analysis of Q-band DEER data acquired for spin-labeled protein A (Q39C-R1p/K88C-R1p) by using validated Tikhonov regularization (n=1000), the protein was fully deuterated while the R1p label was protonated (^2^H-Leu, ^1^H-R1p). In each panel, the top half displays the experimental (blue) and bestfit (red) DEER echo curves; the bottom half shows the corresponding residuals between experimental and calculated curves. The data at each temperature and 2т_2_ combination were fitted individually using validated Tikhonov regulation in the program DeerLab.[[11]](https://paperpile.com/c/IU5B3N/aZSx) The mean normalized χ^2^ value of the fits is given in Table 1 of the main text.

**
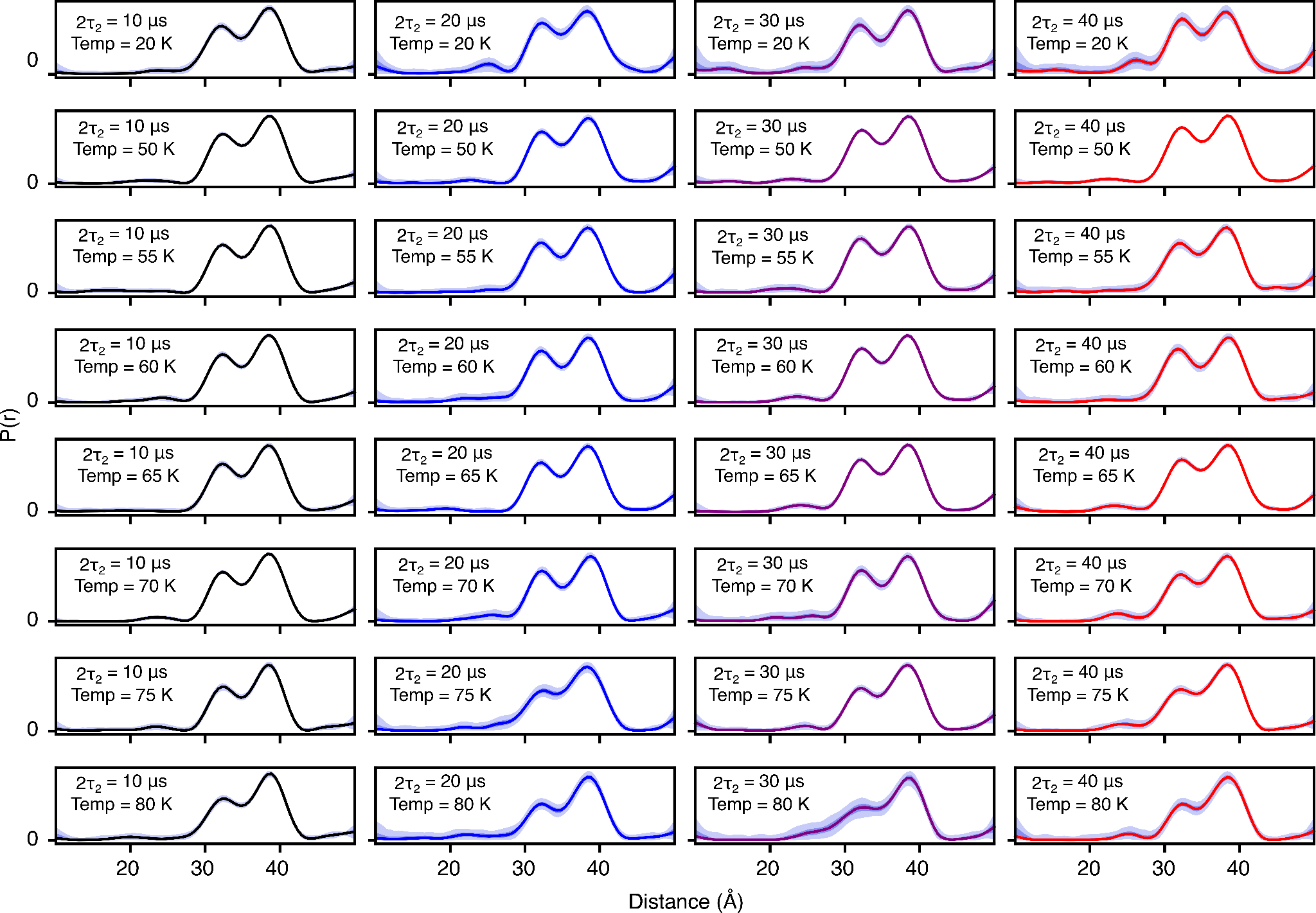
**

**Figure S26.** DEER-derived *P(r)* distributions for spin-labeled protein A (Q39C-R1p/K88C-R1p) by using validated Tikhonov regularization (n=1000), the protein was fully deuterated while the R1p label and the leucine sidechains were protonated (^1^H-Leu, ^1^H-R1). The fits to the experimental DEER echo curves are shown in Fig. S25. The solid lines present 2т_2_ of 10 (*black*), 20 (*blue*), 30 (*lilac*) and 40 μs (*red*). The shaded region below presents the 95% (*light violet*) and 50% (*dark violet*) confidence interval.

**
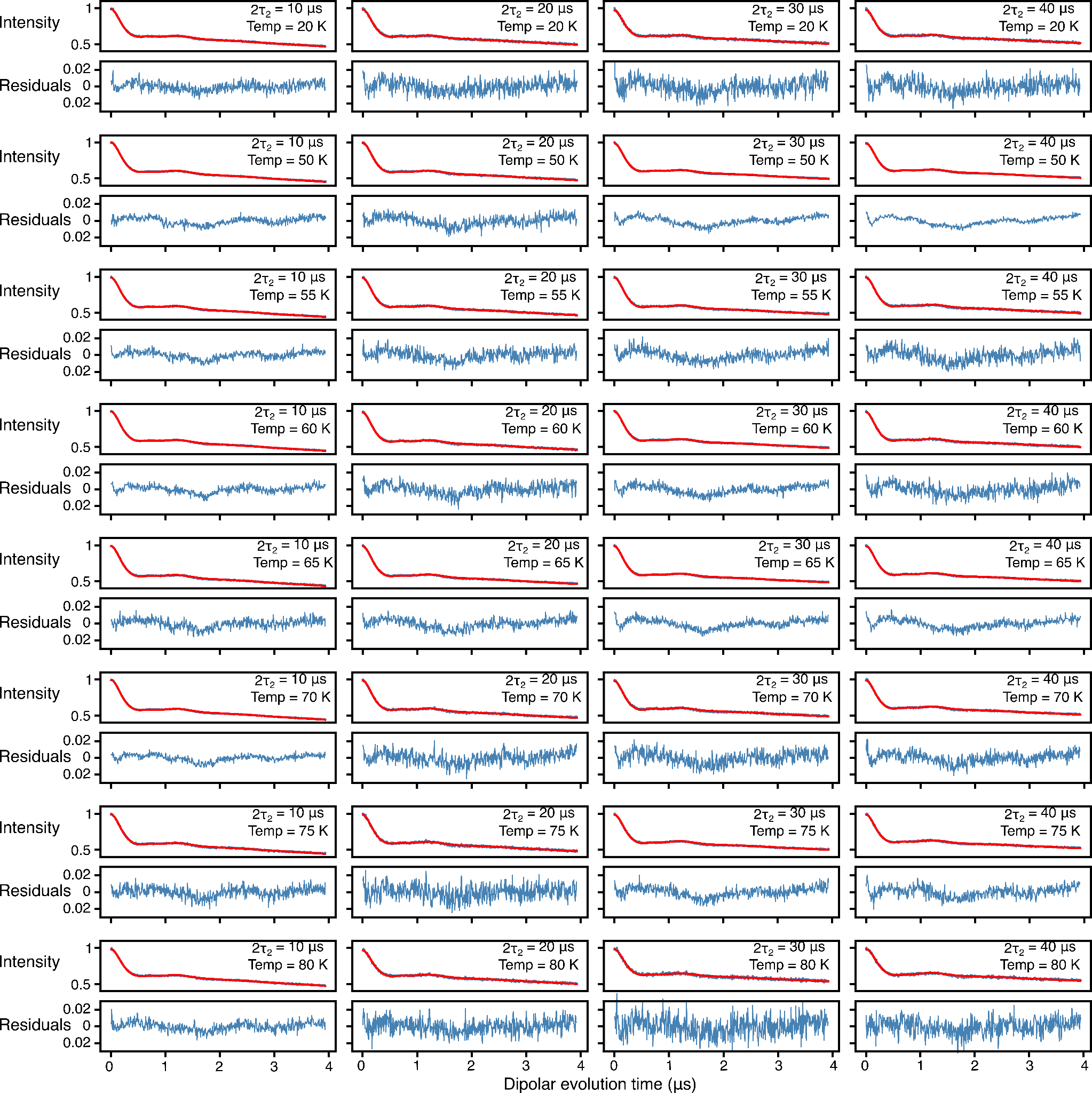
**

**Figure S27.** Global analysis of Q-band DEER data acquired for spin-labeled protein A (Q39C-R1p/K88C-R1p), the protein was fully deuterated while the R1p label was protonated (^2^H-Leu, ^1^H-R1p), using a 2-Gaussian restrained fit. In each panel, the top half displays the experimental (blue) and bestfit (red) DEER echo curves; the bottom half shows the corresponding residuals between experimental and calculated curves. The data at each temperature and 2т_2_ combination were fitted simultaneously with the peak positions and corresponding peak widths in the P(r) distributions treated as global parameters using an in-house Python script [[9,10]](https://paperpile.com/c/IU5B3N/qyEV+JBVW) based on the program DD/GLADDvu.[[12,13]](https://paperpile.com/c/IU5B3N/rCrQ+e6QU) The values of the reduced χ^2^ and optimized global parameters are provided in Table 1 of the main text.

**
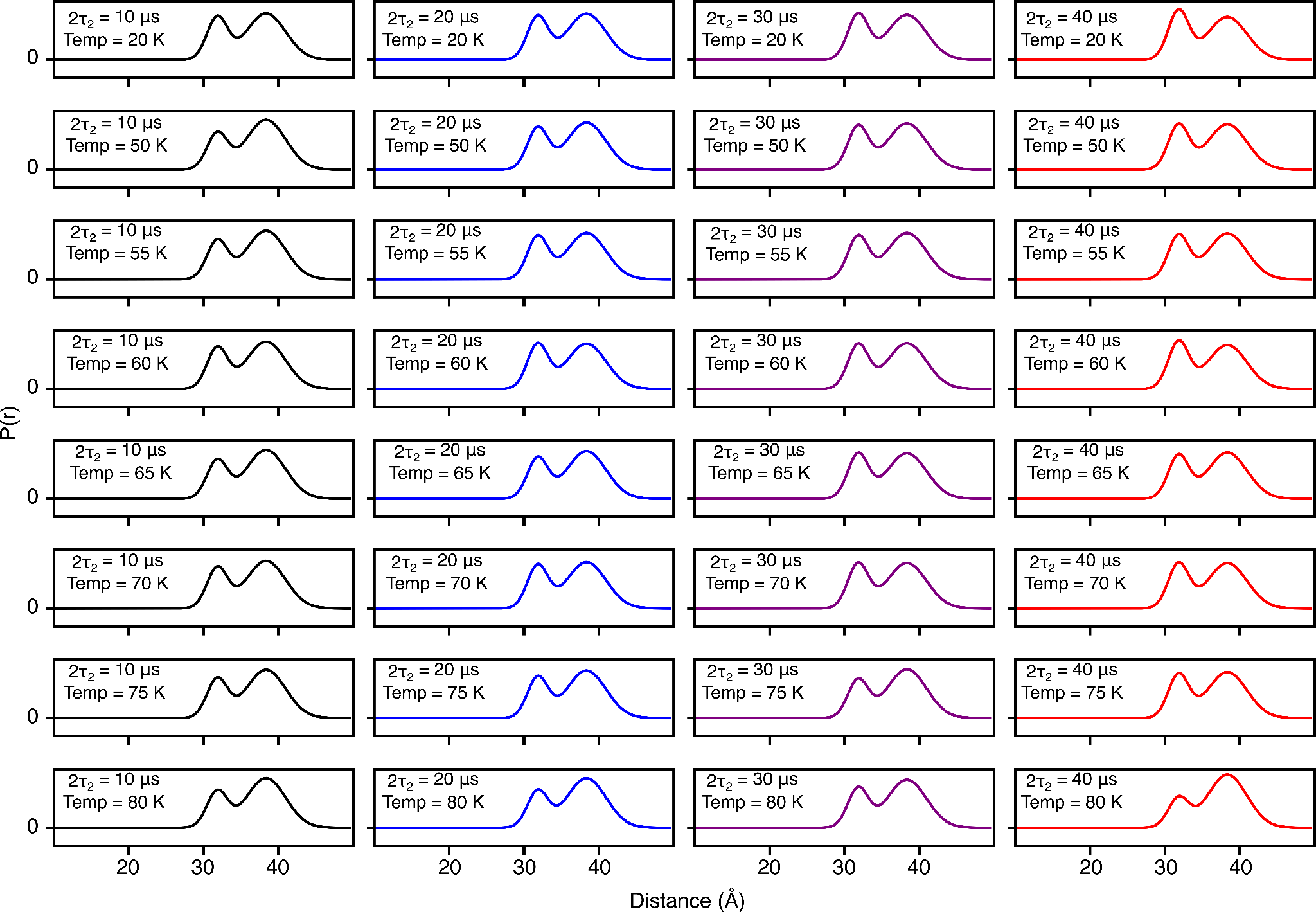
**

**Figure S28.** DEER-derived *P(r)* distributions for spin-labeled protein A (Q39C-R1p/K88C-R1p) by using two-Gaussian global fit in which the peak positions and corresponding widths are treated as global parameters (see main text for details). The protein was fully deuterated while the R1p label was protonated (^2^H-Leu, ^1^H-R1p). The fits to the experimental DEER echo curves are shown in Fig. S27. The solid lines present 2т_2_ of 10 (*black*), 20 (*blue*), 30 (*lilac*) and 40 μs (*red*).

**
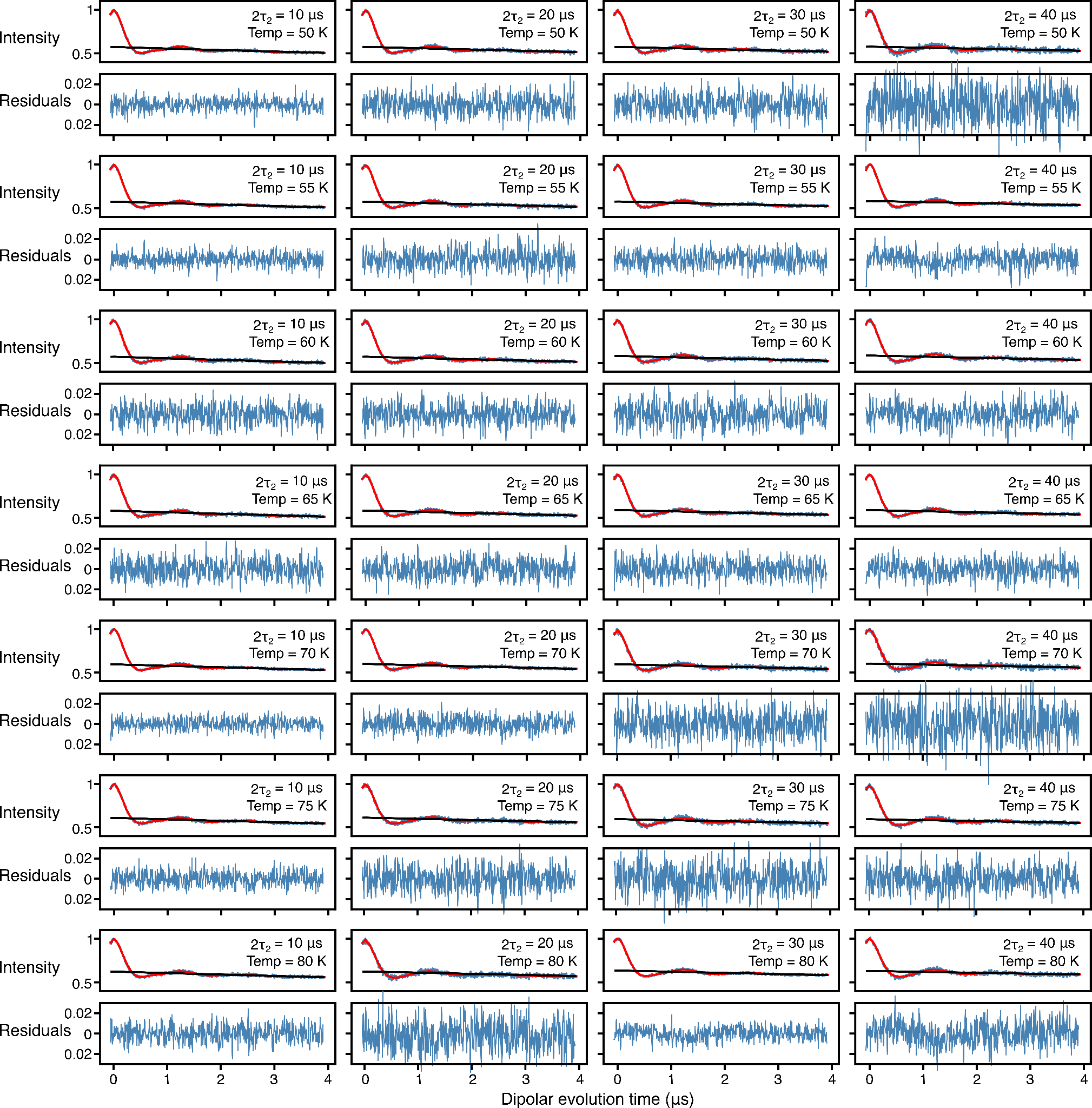
**

**Figure S29.** Analysis of Q-band DEER data acquired for spin-labeled protein A (Q39C-R1p/K88C-R1p), the protein was fully deuterated with site specific leucine protonation while the R1p label was also protonated (^1^H-Leu, ^1^H-R1p), by using validated Tikhonov regularization (n=1000). In each panel, the top half displays the experimental (blue) and bestfit (red) DEER echo curves; the bottom half shows the corresponding residuals between experimental and calculated curves. The data at each temperature and 2т_2_ combination were fitted individually using validated Tikhonov regulation in the program DeerLab.[[11]](https://paperpile.com/c/IU5B3N/aZSx) The mean normalized χ^2^ value of the fits is given in Table 1 of the main text.

**
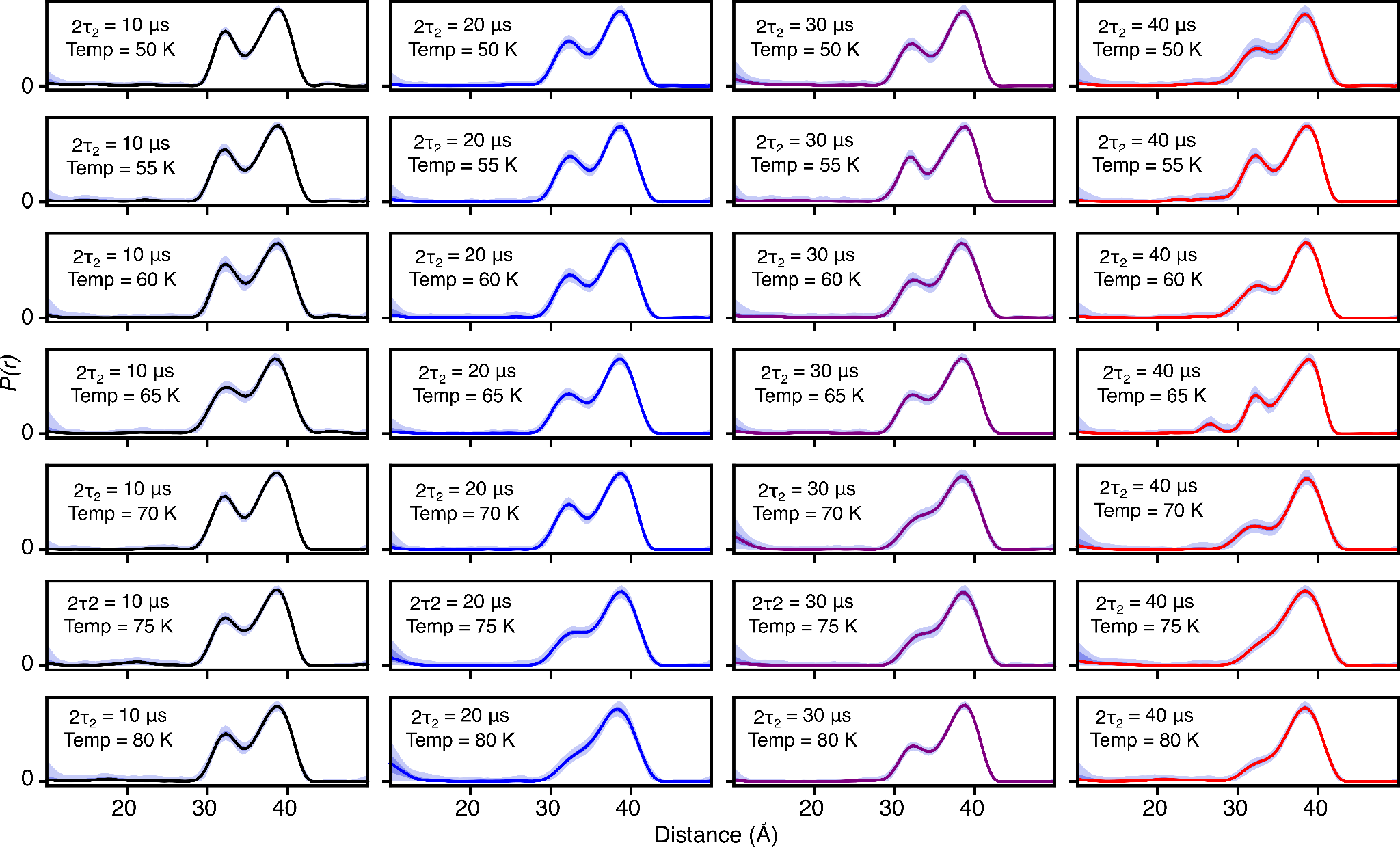
**

**FigureS30.** Analysis of Q-band DEER data acquired for spin-labeled protein A (Q39C-R1p/K88C-R1p) by using validated Tikhonov regularization (n=1000), the protein was fully deuterated while the Leucine side chains and the R1p label were protonated (^1^H-Leu, ^1^H-R1p). In each panel, the top half displays the experimental (blue) and bestfit (red) DEER echo curves; the bottom half shows the corresponding residuals between experimental and calculated curves. The data at each temperature and 2т_2_ combination were fitted individually using validated Tikhonov regulation in the program DeerLab. The mean normalized χ^2^ value of the fits is given in Table 1 of the main text.

**
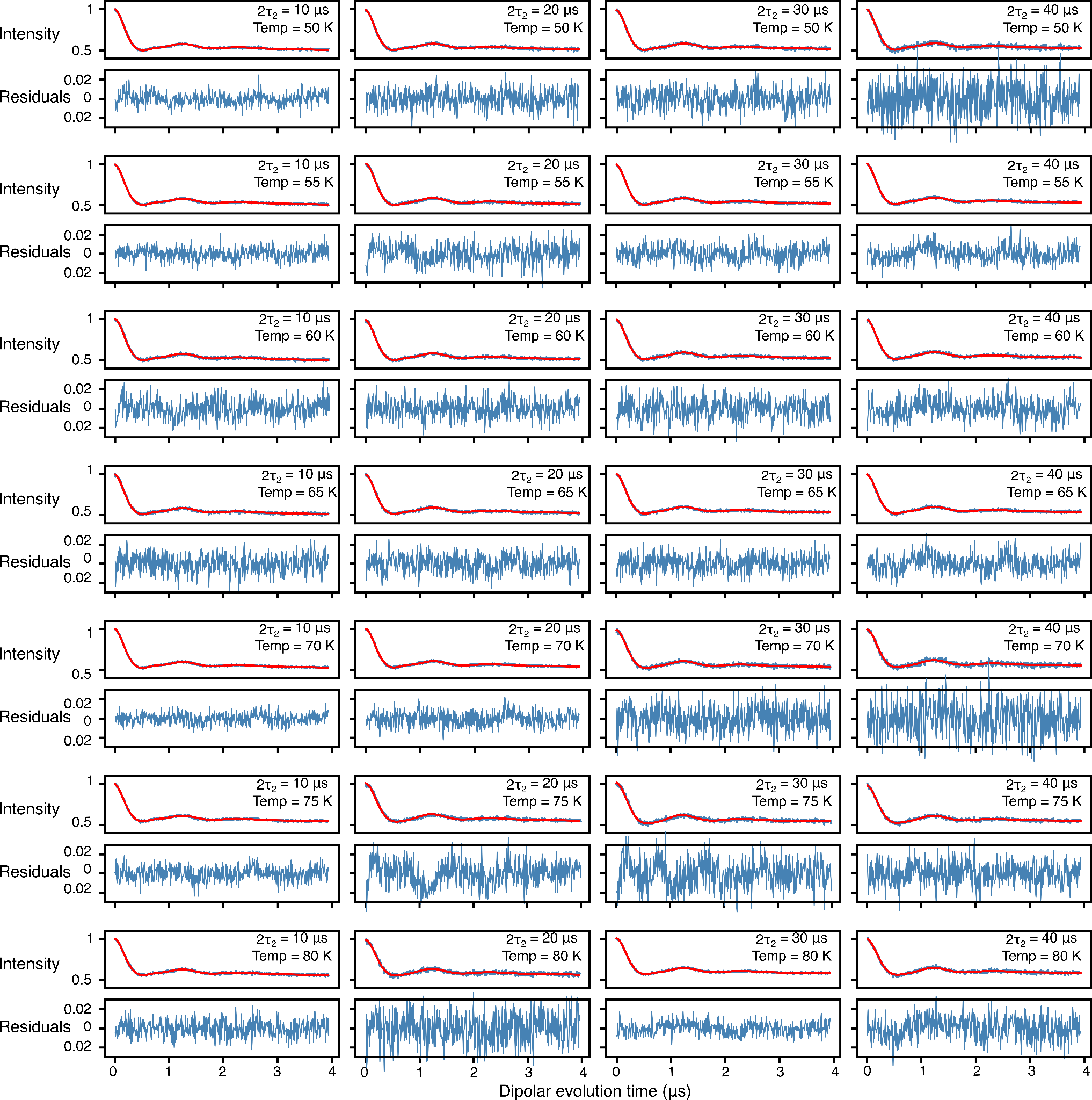
**

**Figure S31.** Global analysis of Q-band DEER data acquired for spin-labeled protein A (Q39C-R1p/K88C-R1p), the protein was fully deuterated with site specific leucine protonation while the R1p label was also protonated (^1^H-Leu, ^1^H-R1p), using a 2-Gaussian restrained fit. In each panel, the top half displays the experimental (blue) and bestfit (red) DEER echo curves; the bottom half shows the corresponding residuals between experimental and calculated curves. The data at each temperature and 2т_2_ combination were fitted simultaneously with the peak positions and corresponding peak widths in the P(r) distributions treated as global parameters using an in-house Python script [[9,10]](https://paperpile.com/c/IU5B3N/qyEV+JBVW) based on the program DD/GLADDvu.[[12,13]](https://paperpile.com/c/IU5B3N/rCrQ+e6QU) The values of the reduced χ^2^ and optimized global parameters are provided in Table 1 of the main text.

**
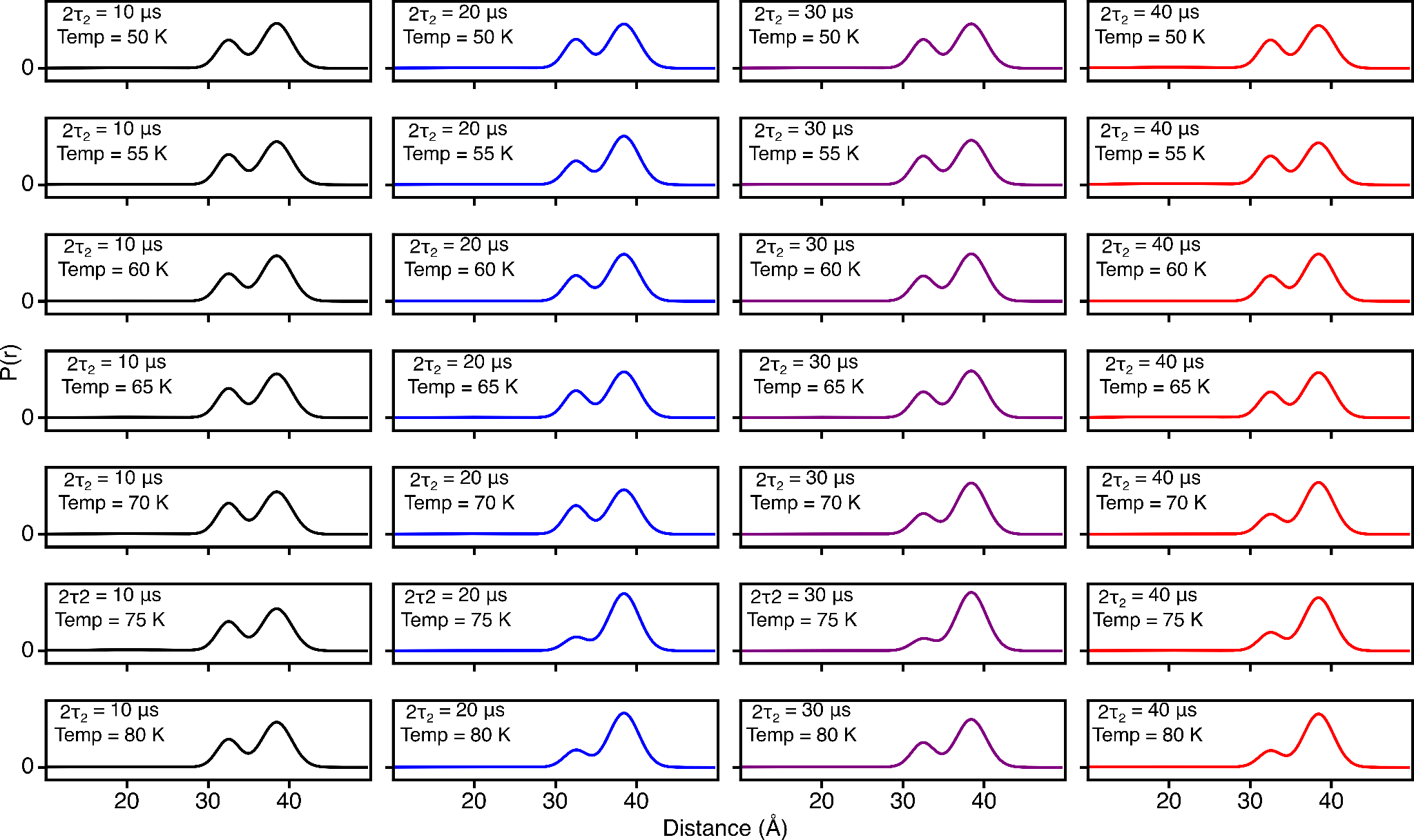
**

**Figure S32.** DEER-derived *P(r)* distributions for spin-labeled protein A (Q39C-R1p/K88C-R1p) by using two-Gaussian global fit in which the peak positions and corresponding widths are treated as global parameters (see main text for details). The protein was fully deuterated while the leucine side chains and R1p label were protonated (^1^H-Leu, ^1^H-R1p). The fits to the experimental DEER echo curves are shown in Fig. S31. The solid lines present 2т_2_ of 10 (*black*), 20 (*blue*), 30 (*lilac*) and 40 μs (*red*).

**Supplementary References**

[1. T. Schmidt and G. Marius Clore, Chemical Communications **56**, 10890 (2020).](http://paperpile.com/b/IU5B3N/2tmD)

[2. V. Tugarinov, V. Kanelis, and L. E. Kay, Nat. Protoc. **1**, 749 (2006).](http://paperpile.com/b/IU5B3N/93p0)

[3. M. Pannier, S. Veit, A. Godt, G. Jeschke, and H. W. Spiess, J. Magn. Reson. **142**, 331 (2000).](http://paperpile.com/b/IU5B3N/6295)

[4. L. K. Nicholson, L. E. Kay, D. M. Baldisseri, J. Arango, P. E. Young, A. Bax, and D. A. Torchia, Biochemistry **31**, 5253 (1992).](http://paperpile.com/b/IU5B3N/GyoE)

[5.](http://paperpile.com/b/IU5B3N/kZwg) L. Vugmeyster, D. Ostrovsky, K. Penland, GL. Hoatson, and RL. Vold, J Phys Chem B. (2013) 117 (4), 1051-1061.

[6. G. Jeschke, Protein Sci. **30**, 125 (2021).](http://paperpile.com/b/IU5B3N/caRR)

[7. G. Hagelueken, R. Ward, J. H. Naismith, and O. Schiemann, Appl. Magn. Reson. **42**, 377 (2012).](http://paperpile.com/b/IU5B3N/cZod)

[8. M. H. Tessmer and S. Stoll, PLoS Comput. Biol. **19**, e1010834 (2023).](http://paperpile.com/b/IU5B3N/Rbxk)

[9. T. Schmidt, J. Jeon, W.-M. Yau, C. D. Schwieters, R. Tycko, and G. M. Clore, Proc. Natl. Acad. Sci. U. S. A. **119**, (2022).](http://paperpile.com/b/IU5B3N/qyEV)

[10. T. Schmidt, D. Wang, J. Jeon, C. D. Schwieters, and G. M. Clore, J. Am. Chem. Soc. **144**, 12043 (2022).](http://paperpile.com/b/IU5B3N/JBVW)

[11. L. Fábregas Ibáñez, G. Jeschke, and S. Stoll, Magn. Reson. **1**, 209 (2020).](http://paperpile.com/b/IU5B3N/aZSx)

[12. S. Brandon, A. H. Beth, and E. J. Hustedt, J. Magn. Reson. **218**, 93 (2012).](http://paperpile.com/b/IU5B3N/rCrQ)

[13. E. J. Hustedt, R. A. Stein, and H. S. Mchaourab, J. Gen. Physiol. **153**, (2021).](http://paperpile.com/b/IU5B3N/e6QU)
